# Wnt Signaling Interacts with Bmp and Edn1 to Regulate Dorsal-Ventral Patterning and Growth of the Craniofacial Skeleton

**DOI:** 10.1371/journal.pgen.1004479

**Published:** 2014-07-24

**Authors:** Courtney Alexander, Sarah Piloto, Pierre Le Pabic, Thomas F. Schilling

**Affiliations:** Department of Developmental and Cell Biology, University of California Irvine, Irvine, California, United States of America; University of Pennsylvania School of Medicine, United States of America

## Abstract

Craniofacial development requires signals from epithelia to pattern skeletogenic neural crest (NC) cells, such as the subdivision of each pharyngeal arch into distinct dorsal (D) and ventral (V) elements. Wnt signaling has been implicated in many aspects of NC and craniofacial development, but its roles in D-V arch patterning remain unclear. To address this we blocked Wnt signaling in zebrafish embryos in a temporally-controlled manner, using transgenics to overexpress a dominant negative Tcf3, (dntcf3), (*Tg(hsp70I:tcf3-GFP*), or the canonical Wnt inhibitor dickkopf1 (dkk1), (*Tg(hsp70i:dkk1-GFP*) after NC migration. In dntcf3 transgenics, NC cells in the ventral arches of heat-shocked embryos show reduced proliferation, expression of ventral patterning genes (*hand2*, *dlx3b*, *dlx5*a, *msxe*), and ventral cartilage differentiation (e.g. lower jaws). These D-V patterning defects resemble the phenotypes of zebrafish embryos lacking Bmp or Edn1 signaling, and overexpression of dntcf3 dramatically reduces expression of a subset of Bmp receptors in the arches. Addition of ectopic BMP (or EDN1) protein partially rescues ventral development and expression of *dlx3b*, *dlx5a*, and *msxe* in Wnt signaling-deficient embryos, but surprisingly does not rescue *hand2* expression. Thus Wnt signaling provides ventralizing patterning cues to arch NC cells, in part through regulation of Bmp and Edn1 signaling, but independently regulates *hand2*. Similarly, heat-shocked dkk1+ embryos exhibit ventral arch reductions, but also have mandibular clefts at the ventral midline not seen in dntcf3+ embryos. Dkk1 is expressed in pharyngeal endoderm, and cell transplantation experiments reveal that dntcf3 must be overexpressed in pharyngeal endoderm to disrupt D-V arch patterning, suggesting that distinct endodermal roles for Wnts and Wnt antagonists pattern the developing skeleton.

## Author Summary

Craniofacial abnormalities are among the most common birth defects. Understanding the molecular mechanisms underlying craniofacial disorders is crucial for developing treatment strategies. Much of the craniofacial skeleton arises from specialized embryonic structures known as pharyngeal arches. Patterning of these arches requires precise spatial and temporal expression of multiple genes, which is coordinated between tissues by secreted signals. Wnts are secreted ligands expressed throughout the pharyngeal arches yet their role in craniofacial patterning remains unclear. In this study we examine the role of Wnts in craniofacial patterning using transgenic zebrafish to inhibit downstream Wnt signaling. We show that Wnt signaling deficient embryos have lower jaw specific defects, which strongly resembles loss-of-function phenotypes in both the Bmp and Edn1 signaling pathways. Through rescue experiments we find that Wnts are upstream regulators of both Bmp and Edn1 signaling. We thus have uncovered a crucial requirement for Wnt signaling in craniofacial patterning.

## Introduction

A fundamental question in skeletal biology is how cartilages and bones with distinct shapes arise from skeletogenic precursor cells. Much of the craniofacial skeleton derives from neural crest (NC) cells that migrate in streams into the pharyngeal arches and contain anterior-posterior (A-P) patterning information obtained prior to migration [1]–[3]. However, these NC cells also become intimately associated with epithelia, including surface ectoderm and pharyngeal endoderm, which produce signals important for skeletal patterning. For example, Fgf8 from the facial ectoderm regulates A-P polarity of the mandibular arch as well as NC proliferation/survival [4]–[6]. Surgical disruption of the pharyngeal endoderm in chick [7], or mutations that disrupt endoderm in zebrafish, lead to severe cartilage malformations [8]–[10]. Endoderm-derived Fgf3 induces cartilage formation [9] and sphingosine phosphate-1 from endoderm modulates Shh signaling to promote mandibular growth and patterning [11]–[13]. Ectoderm-derived Shh induces upper jaw and neurocranial structures [14], [15]. Thus, craniofacial skeletal shapes reflect interplay between epithelial signals and intrinsic properties of mesenchyme, but the mechanisms underlying these interactions remain unclear.

One well-studied example of such epithelial-mesenchymal interactions in the pharyngeal skeleton is the induction of ventral skeletal fates along the dorsal-ventral (D-V) axis of the mandibular arch by ectodermal Endothelin 1 (Edn1) and Bone morphogenetic proteins (Bmps) [16]–[22]. Conditional loss of Bmp4 in the facial ectoderm in mice inhibits ventral mandibular growth and patterning [23]. Loss of Edn1 and/or any of several components of its signal transduction pathway leads to severe jaw truncations, both in mice and in humans, and in some cases transformations of ventral tissues (the lower jaw) to a more dorsal identity [22], [24]–[27]. Initially both Edn1 and Bmps induce similar subsets of ventral/intermediate genes as well as restricting Jag1 signaling to the dorsal domain [16], [19], [20], [28]. But once arch primordia are established, effects of Bmps become more ventrally restricted to domains that no longer depend on Edn1, particularly the transcription factor Hand2. Uniform application of Bmp or Edn1 proteins can restore many aspects of D-V patterning in Bmp- and Edn1-deficient zebrafish embryos, suggesting that other ventralizing signals must interact to control D-V patterning [22].

Wnts are good candidates for additional signals involved in D-V patterning based on their localized expression and known requirements in craniofacial development. Several Wnt ligands show restricted expression in facial epithelia (ectoderm, endoderm) in zebrafish, chick and mouse embryos [29]–[32] and Frizzled (Fzd) receptors are expressed throughout arch NC cells and endoderm [31], [33], [34]. In addition, expression of Lef1, Tcf1, β-catenin (βcat), [35], and transgenic Wnt signaling reporter lines (Lef/Tcf promoters driving β-gal or LacZ expression) in mice all are ventrally (distally) restricted in the mandibular as well as distal maxillary prominences [36], [37]. Like Bmps, Wnt signaling is necessary for early NC cell induction [38], [39] and also plays later roles in NC migration, fate specification, and proliferation [40]. In zebrafish, conditional overexpression of a dominant negative Tcf3 (dntcf3) during NC cell specification dramatically reduces NC cell numbers [41], similar to depletion of Fzd3 receptors in Xenopus [42]. *Wnt1^−/−^/Wnt3^−/−^* double mutant mice show reduced proliferation of pre-migratory NC cells [43]. Conditional loss of βcat in the pharyngeal ectoderm impairs growth of the facial prominences [44], while conditional loss of βcat in cranial NC cells leads to apoptosis and a nearly complete loss of NC-derived craniofacial structures [45]. Finally, loss of Tcf4/Lef1 function or overexpression of the Wnt inhibitor Dickkopff-1 (Dkk1) results in smaller facial structures and clefting between the frontonasal and maxillary prominences [36]. Similarly, Wnt signaling is important for facial midline development in humans as incidences of cleft lip and palate have been mapped to genetic variations in Wnt ligands [46].

In this study, we examine temporal requirements for Wnt signaling in zebrafish D-V craniofacial development. We utilize two transgenic lines, *Tg(hsp701:dkk1-GFP)*(dkk1+) and *Tg(hsp70I:tcf3-GFP)* (dntcf3+), to interfere with Wnt signaling conditionally, in a stage-specific manner. *Tg(hsp701:dkk1-GFP)* embryos overexpress dkk1, a secreted negative regulator, while *Tg(hsp70I:tcf3-GFP)* embryos overexpress a dominant negative form of the Tcf3 transcription factor. Both methods of inhibiting Wnt signaling after NC cell migration result in proliferation and ventral patterning defects in the mandibular and hyoid arches. Interestingly, dkk1+ embryos also show unique clefting of the lower jaw. Defects in ventral-intermediate specific gene expression and expansion of the dorsal specific *jag1b* resemble loss of Bmp and Edn1 signaling [19], [20]. We show that Wnt signaling promotes Bmp signaling through regulation of expression of two specific Bmp receptors, *bmpr1ab* and *bmpr1ba*, in the pharyngeal arches. Ectopic Bmp protein can rescue *msxe*, *dlx3b*, *dlx5a* but not *hand2* in the absence of Wnt signaling, demonstrating that Wnts participate in a regulatory network with Bmp and Edn1 signaling, but separately in *hand2* regulation, to control D-V pharyngeal patterning. Chimeric analyses reveal that dntcf3 acts cell autonomously in pharyngeal endoderm, which also expresses *dkk1*. This suggests that Wnts regulate patterning in the endoderm, which through some as yet unknown signal imparts D-V patterning upon neighboring skeletal progenitors in the NC.

## Results

### Wnt responses are highest in the ventral pharyngeal arches

**Figure 1:**
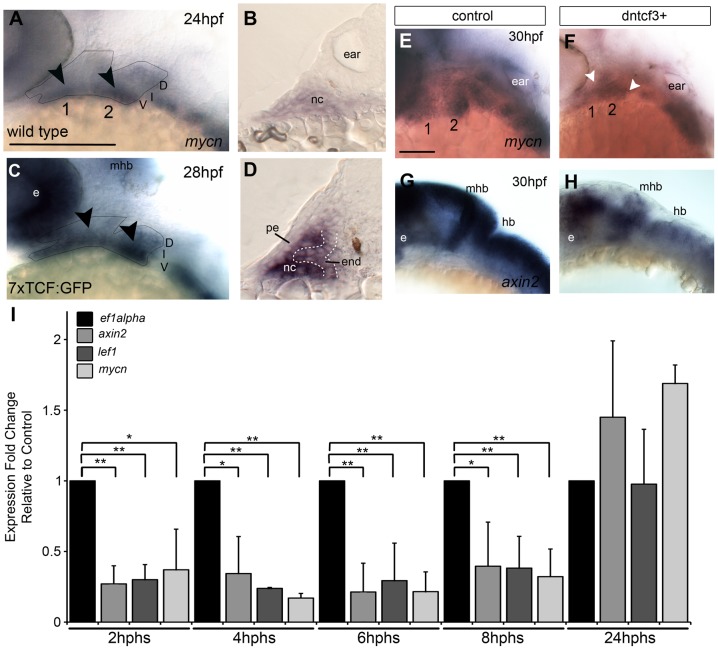
Wnt signaling in the pharyngeal arches. (A–H) In situ hybridization (ISH) and (I) quantitative, real-time PCR (qPCR) analysis of Wnt target gene expression; (A, C, E–H) lateral views, anterior to the left; (B, D) transverse sections through arch 2. (A, B) *mycn* mRNA is ventrally restricted (black arrowheads) in wild type (WT) embryos. Arches 1 and 2 are outlined by dotted lines. (C, D) *GFP* mRNA is ventrally restricted (arrowheads) in *Tg(7xTCF-Xla.Siam:GFP)^ia4^* transgenics. (B, D) Both *mycn* (B) and 7xTCF;GFP (D) are expressed in ventral nc cells and pharyngeal endoderm, and excluded from pharyngeal ectoderm. (E–H) *mycn* and *axin2* expression in controls (E, G), and dntcf3+ embryos at 26 hpf (F, H; heat shocked at 22 hpf). (I) qPCR analysis of *axin2*, *lef1* and *mycn* expression in dntcf3+ embryos, normalized to nontransgenic, heat-shocked controls, with *ef1alpha* as an internal control. * P<0.05, ** P<0.001. Abbreviations: e, eye; D, dorsal arch; end, pharyngeal endoderm; hb, hindbrain; I, intermediate arch; mhb, mid-hindbrain boundary; nc, neural crest; pe, pharyngeal ectoderm; V, ventral arch. Scale bars: 100 µm.

Numerous Wnt ligands (Wnt2, Wnt4, Wnt5a/b, Wnt 6, and Wnt7a/b) and receptors (Fzd1, Fzd3, Fzd4, Fzd6, Fzd7, Fzd8, and Fzd10) are expressed broadly in the pharyngeal ectoderm, endoderm, neural crest (NC), and mesoderm [29], [32], [34], [35]. To determine which regions of the pharyngeal arches respond directly to Wnt signaling we used in situ hybridization (ISH) to examine expression of the direct downstream Wnt target *mycn* (Fig. 1A,B), an oncogene with roles in regulating Wnt-dependent morphogenesis and proliferation [47], [48]. *mycn* mRNA was detected throughout the arches but at higher levels in the ventral domain, primarily within the NC mesenchyme (arrowheads in Fig. 1A,B). To further address which pharyngeal tissues respond directly, we examined expression of a transgenic Wnt reporter zebrafish *Tg(7xTCF-Xla.Siam:GFP)^ia4^* (7xTCF:GFP) [49], which contains seven TCF response elements driving expression of GFP, thus acting as a live reporter in cells where stabilized β-catenin (βcat) interacts with Tcf transcription factors. ISH for GFP mRNA at 28 hours postfertilization (hpf) revealed regions of 7xTCF:GFP expression in the ventral first and second arches (Fig. 1C), which in transverse sections appeared localized both to arch NC cells and pharyngeal endoderm, but not pharyngeal ectoderm (Fig. 1D).

### Blocking Wnt signaling after NC migration disrupts the ventral arch skeleton

To bypass earlier requirements for Wnts in embryogenesis we took a conditional loss-of-function approach using heat shock-inducible transgenic zebrafish lines to inhibit Wnt signaling in a temporally-controlled manner. *Tg(hsp70I:tcf3-GFP)* (hs-dntcf3) embryos overexpress a truncated form of the transcription factor *tcf3* with GFP replacing the βcat-interacting domain, under control of heat shock promoter 70 [41]. With a similar hsp70 promoter, *Tg(hsp701:dkk1-GFP)* (hs-dkk1) embryos overexpress full length *dkk1b* tagged with a GFP [50], which prevents Fzd-Lrp co-receptor binding [51], [52].

To verify that Wnt-βcat signaling was affected in hs-dkk1+ and hs-dntcf3+ embryos we used ISH to examine the expression of *mycn* and *axin2*, both direct Wnt targets, after heat shocking during stages of craniofacial patterning. At 4 hours post heat shock (hphs) hs-dntcf3+ embryos heat shocked at 22 hpf showed severe reductions in expression of *mycn* in the arches, eyes, and brain and *axin2* (which shows only very weak or no expression in pharyngeal arches), in the eyes and brain (Fig. 1E–H). Similarly, compared with controls (Fig. S1A, C) at 2 hphs hs-dkk1+ embryos heat shocked at 24 hpf showed reduced *mycn* in the arches, eye, and brain and *axin2* expression in the brain but to a lesser extent than in hs-dntcf3+ embryos (Fig. S1B, D).

To determine stage-specific defects caused by disrupting Wnt signaling in hs-dntcf3+ and hs-dkk1+ embryos we performed quantitative real time PCR (qPCR) analysis of direct Wnt targets. In hs-dntcf3+ embryos, *axin2* and *mycn* expression was reduced significantly from 2–8 hphs (Fig. 1I), but recovered and slightly increased by 24 hphs (Fig. 1I). *Lef1*, a transcriptional cofactor in the Wnt pathway [53], [54], was also significantly reduced in hs-dntcf3+ embryos from 2–8 hphs (Fig. 1I). In hs-dkk1+ embryos, *axin2* expression was severely reduced from 1–8 hphs, while *Lef1* downregulation started later, from 4–8 hphs (Fig. S1E). Thus both hs-dntcf3 and hs-dkk1 lines deplete Wnt signaling almost immediately after heat shock and repress Wnt signaling for up to 8 hphs.

**Figure 2:**
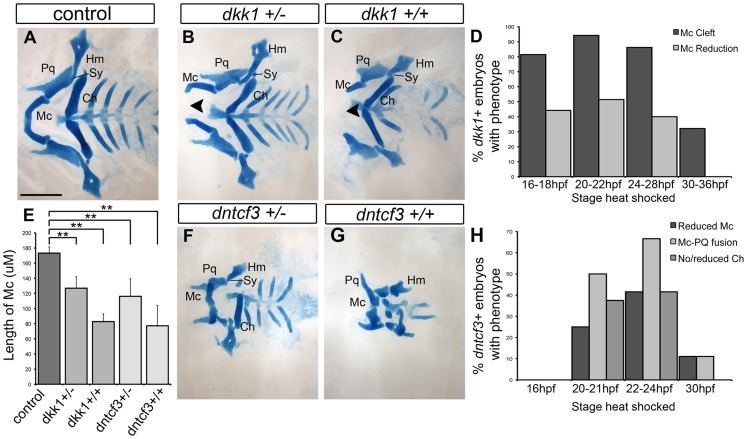
Requirements for Wnt signaling in craniofacial cartilage development. (A–C, F, G) Alcian blue stained cartilage at 96 hpf, dissected and flat mounted, ventral views, anterior to the left. (A) Wild-type control, (B) dkk1^+/−^ heterozygote, (C) dkk1^+/+^ homozygote, (F) dntcf3^+/−^ heterozygote, (G) dntcf3^+/+^ homozygote, heat shocked as described (see Results). Arrowheads indicate clefting of Meckel's cartilage (Mc). (D) Histogram quantifying the frequency of Mc defects in dkk1+ embryos heat shocked at different stages from 16–36 hpf. (E) Histogram quantifying average Mc length, p<0.01. (H) Histogram quantifying the frequency of cartilage defects in arches 1 and 2 in dntcf3+ embryos heat shocked at different stages from 16–30 hpf. Abbreviations: Ch, ceratohyal; Hm, hyomandibular; Mc, Meckels; Pq, palatoquadrate; Sy, symplectic. Scale bar: 100 µm.

Alcian blue staining of cartilage in larvae at 96 hpf revealed that, compared with controls, hs-dkk1+/− heterozygotes heat shocked for 30 min at 16–28 hpf developed mandibular clefting and reduced Meckel's cartilages (Mc), as well as mild reductions in other craniofacial cartilages (Fig. 2A, B, D). Homozygous hs-dkk1+/+ larvae displayed dramatic shortening of Mc ventrally in the first arch, as well as the symplectic (Sy), a more intermediate/dorsal element of the second arch (Fig. 2C, E; [19], [20]). Thus, hereafter “dkk1+” refers to homozygous hs-dkk1 embryos/larvae heat shocked for 30 min between 20–22 hpf.

Heterozygous hs-dntcf3+ larvae heat shocked slightly later (22–24 hpf) also showed mild reductions in Mc, but in this case Mc was fused to the more dorsal palatoquadrate (Pq) in arch 1 (Fig. 2F, H). The ceratohyal (Ch) in ventral arch 2 was also variably reduced, but more posterior cartilages appeared largely unaffected. Cartilage defects in homozygous hs-dntcf3 larvae heat shocked similarly were much more severe (Fig. 2E, G) – 41% showed reduced Mc and Ch reduction/loss, while 66% showed joint fusion between Mc and Pq (Fig. 2H). Thus, hereafter “dntcf3+” refers to heterozygous hs-dntcf3+/− embryos/larvae heat shocked for 12 min between 22–24 hpf.

To determine tissue-specific requirements for Wnt signaling in the pharyngeal arches, we transplanted dntcf3+ cells at gastrula stages either into the fate map position that gives rise to NC or co-injected with Taram-A mRNA to drive them to an endodermal fate, into non-transgenic WT hosts [9]. While dntcf3+ NC cells in chimeras that virtually filled the entire mandibular arch caused no discernable cartilage defects (not shown), large grafts of dntcf3+ endodermal cells into the pharyngeal region induced D-V patterning defects that resembled dntcf+ embryos, including reduced Mc and fused Mc-Pq (Fig. S2A). These results suggest that the critical direct response to Wnt occurs in the endoderm (which expresses 7XTCF:GFP) and is indirectly relayed to surrounding NC cells.

To verify that cartilage defects in dkk1+ and dntcf3+ larvae reflect specific requirements for Wnt signaling we attempted to rescue them using the compound 6-bromoindirubin-3′-oxime (BIO), which stabilizes Wnt signaling by inhibiting GSK-3 [55]. BIO treatments of 7xTCF:GFP embryos at 24 hpf caused ectopic *gfp* expression and direct Wnt targets were upregulated in a dose-dependent manner as determined by ISH and qPCR analysis at 30 hpf (Fig. S3). Treatment of wild type embryos with BIO resulted in an overall reduction of cartilages in a dose-dependent manner, which correlated with reduced proliferating cell nuclear antigen (*pcna*) expression (which marks cells in mitosis [56]) in the arches at 30 hpf, indicating reduced proliferation of cartilage precursors (Fig. S4J–L). Despite their smaller sizes, dorsal cartilages acquired more rod-like morphologies similar to ventral Mc and Ch, suggesting partial ventralization (Fig. S4A–C). BIO treatments of both heat shocked dntcf3+ and dkk1+ transgenics partially rescued cartilage defects, including Mc-Pq joint fusions and Ch was consistently restored in dntcf3+ larvae (Fig. S4D–I, M). BIO also rescued Mc clefting in dkk1+ larvae at higher concentrations (Fig. S4G–I). Therefore loss of canonical Wnt signaling in dntcf3+ and dkk1+ embryos accounts for the majority of craniofacial defects.

### Blocking Wnt signaling disrupts proliferation in ventral arch NC cells

**Figure 3:**
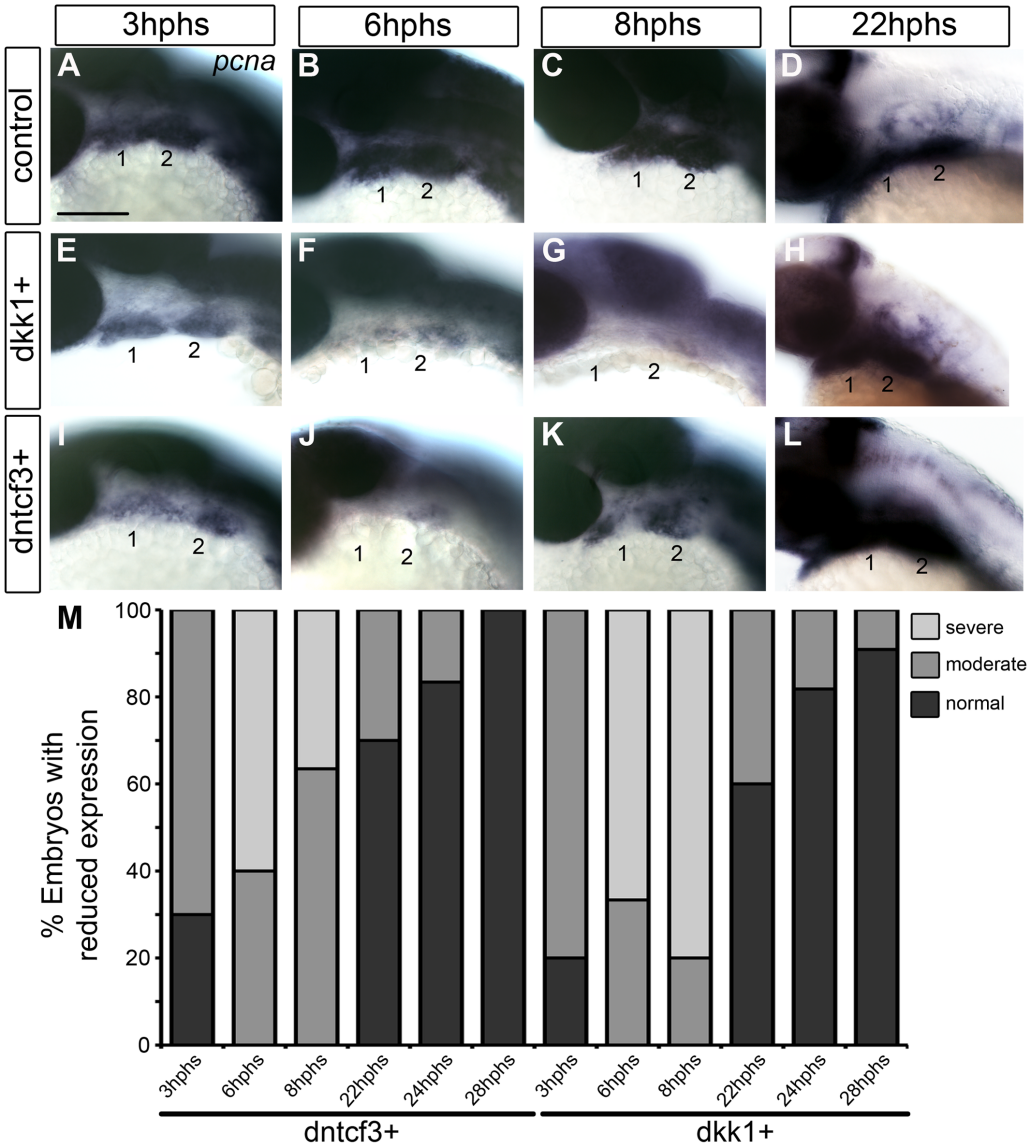
Requirements for Wnt signaling in pharyngeal arch cell proliferation. (A–L) Whole mount ISH for *pcna* in embryos fixed at 3–22 hours post-heat shock (hphs), lateral views, anterior to the left (all heat shocked at 22 hpf). (A–D) *pcna* is expressed throughout arches, brain and eyes. (E–H) dkk1+ embryos have reduced *pcna* expression at 3 hphs (E), severe reductions at 6 hphs (F) and virtually no arch expression at 8 hphs (G), before expression returns at 22 hphs (H). (I–L) dntcf3+ embryos show reduced *pcna* expression at 3 hphs (I), and virtually no expression at 6 hphs (J, K) before expression rebounds between 8–22 hphs (L). (M) Histogram quantifying percentages of dkk1+ and dntcf3+ embryos with moderate versus severe reductions in *pcna* expression. Scale bar: 100 µm.

Cartilages in dntcf3+ and dkk1+ larvae were 30–50% smaller than controls (Fig. 2). This reduced cartilage size was not due to increased cell death as we could detect no differences in the number of acridine orange stained cells in the arches between dntcf3+, dkk1+ and control embryos at 6 hphs (Fig. S5). To examine proliferation in the arches we performed ISH for *pcna*. *Pcna* mRNA was detected throughout the pharyngeal arches from 3–22 hphs (25–44 hpf) in controls (Fig. 3A–D), but somewhat reduced at 3 hphs (25 hpf) in both dkk1+ and dntcf3+ embryos (Fig. 3A, E, I, M). By 6 hphs (28 hpf) *pcna* expression was nearly undetectable in the arches in both dkk1+ (66%, *n* = 6) and dntcf3+ (60%, *n* = 10) (Fig. 3B, F, J, M). By 8 hphs (30 hpf), *pcna* expression had recovered slightly in dntcf3+ embryos (36%, *n* = 11) (Fig. 3K, M) but not in dkk1+ embryos (80%, *n* = 5) (Fig. 3G, M). Both recovered completely by 22–28 hphs (44 hpf) (Fig. 3D, H, L, M).

To confirm these apparent defects in proliferation we used an antibody that recognizes phosphoHistone3 (pH 3), a protein involved in chromosome condensation in mitotic cells [57], which marks a subset of pcna+ dividing cells. In dkk1+ and dntcf3+ embryos pH 3 staining was reduced throughout the eye and brain (Fig. S6A–B, D–E). At 4 hphs, dkk1+ embryos had a 75% reduction in pH 3+ cells in the arches compared to controls (Fig. S6A′, B′, C). Similarly, at 3 hphs dntcf3+ embryos had approximately 50% fewer pH 3+ cells in the arches than controls (Fig. S6D′, E′, F). Thus depleting Wnt signaling in embryos between 24–30 hpf severely impairs proliferation in the arches, which correlates with reductions in cartilage and in Wnt target gene expression (Figs. 1, 2).

### Blocking Wnt signaling disrupts expression of ventral arch patterning genes

**Figure 4:**
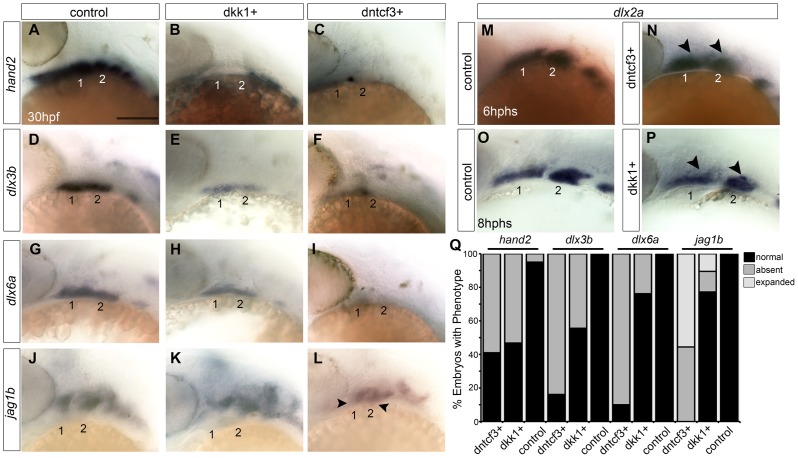
Requirements for Wnt signaling in dorsal-ventral arch patterning. (A–P) Whole mount ISH for genes involved in dorsal-ventral (D-V) patterning in control (A, D, G, J), dkk1+ embryos heat shocked at 20–22 hpf (B, E, H, K), and dntcf3+ embryos heat shocked at 22–24 hpf (C, F, I, L), lateral views, anterior to the left. *hand2* (B, C), *dlx3b* (E, F), and *dlx6a* (H, I) expression is reduced, while *jag1b* (K, L) expression expands ventrally (arrowheads) particularly in dntcf3+ embryos, compared with controls. (M–P) *dlx2a* expression in control (M,O), dntcf3+ (N), and dkk1+ (P) embryos. The dorsal boundary of *dlx2a* expression (arrowheads) appears to shift ventrally in some cases, (N,P) compared to the control (M,O). (Q) Histogram quantifying percentages of dkk1+ and dntcf3+ embryos with defects in D-V patterning. Scale bar: 100 µm.

To investigate roles for Wnt signaling in D-V patterning within the arches, we examined expression of genes that mark distinct ventral, intermediate and dorsal regions of the arch primordia in dntcf3+ and dkk1+ embryos with ISH [19]. *hand2* expression in the ventral-most domains of each arch was severely reduced in both dkk1+ (53%, *n* = 15) and dntcf3+ (59%, *n* = 17) embryos (Fig. 4A–C, Q), with a small domain of expression remaining at the arch 1–2 boundary in dntcf3+ embryos (Fig. 4C). Similarly, expression of *dlx3b* and *dlx6a* in the intermediate domains of each arch were mildly reduced in dkk1+ (44%, *n* = 18, 23%, *n* = 21) and severely reduced in dntcf3+ embryos (83%, *n* = 12; 90%, *n* = 21) (Fig. 4D–I, Q). Finally, expression of the Notch ligand, *jag1b* in the dorsal-most domains of each arch [28], was variably expanded ventrally in dkk1+ (10.5%, *n* = 57) embryos and consistently expanded in dntcf3+ embryos (55.5%, *n* = 9) as well as chimeras in which dntcf3+ cells were transplanted into the pharyngeal endoderm (Fig. 4J–L, Q; Fig. S2B–C). These gene expression changes were not simply due to an overall loss of arches or NC cells, since *dlx2a* expression (Fig. 4N, P) as well as *sox10:lynTdtom* expression throughout the D-V extent of the arch NC were unaffected in the arches of both dkk1+ and dntcf3+ embryos (Fig. S7). Additionally, BIO treatments of wild type embryos caused dorsal expansion of expression of the ventral-intermediate gene *msxe*, mild expansion of *dlx3b* and *hand2* expression, and reduced *jag1b* expression in the dorsal domain (Fig. S8). Therefore, Wnt signaling promotes ventral and intermediate-cell fates in the arches. Dntcf3+ embryos in particular, with residual *hand2* expression at the arch 1–2 boundary, closely resemble mutants in Bmp and Edn1 signaling [19], [20].

### Blocking Wnt signaling disrupts Bmp and Edn1 signaling in the arches

**Figure 5:**
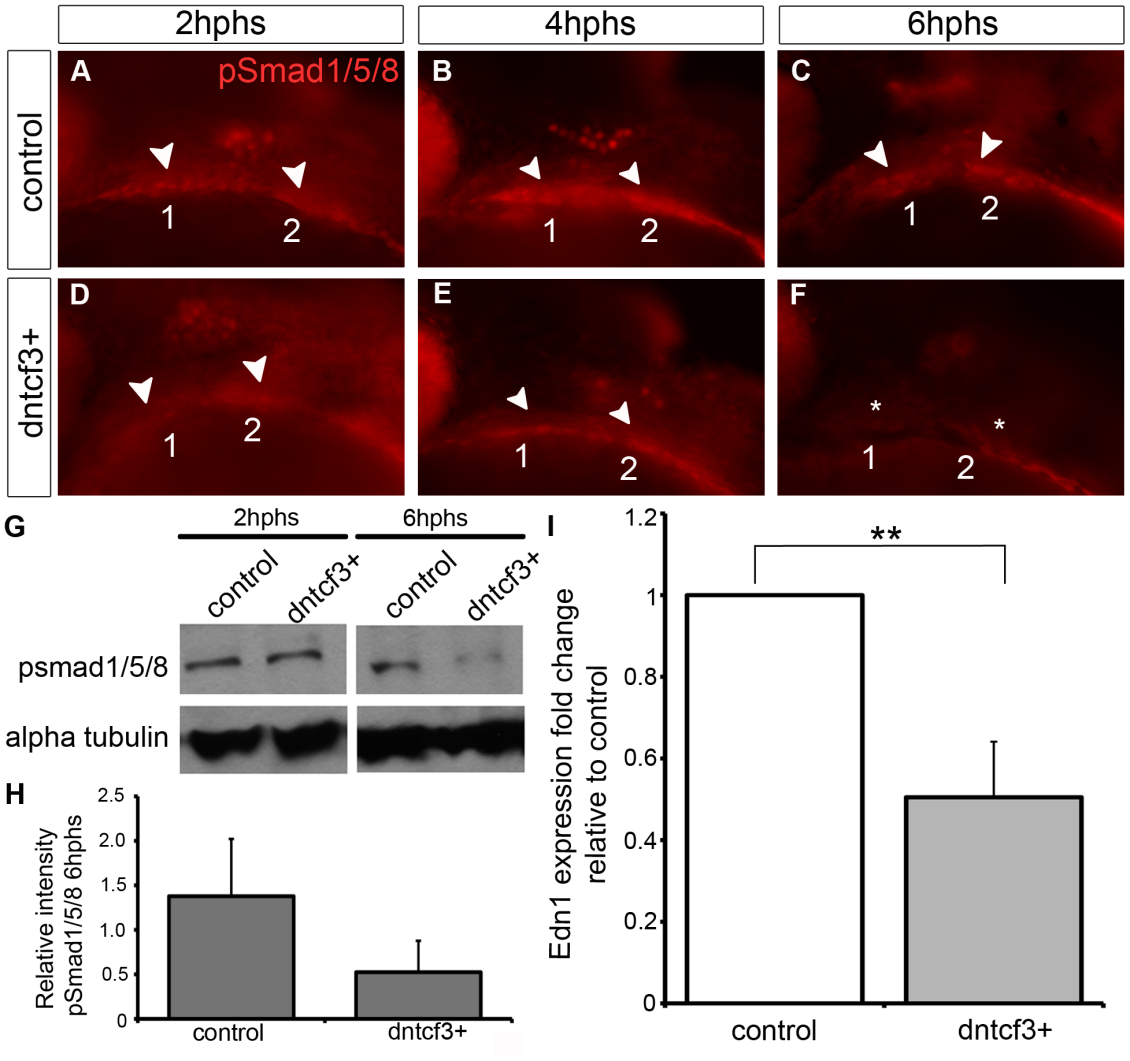
Wnt signaling regulates Bmp and Edn1 signaling in the arches. (A–F) Anti-pSmad1/5/8 staining in control (A–C) and dntcf3+ (D–F) embryos heat shocked at 22–24 hpf, lateral views, anterior to the left. pSmad1/5/8 expression is localized ventrally in the arches (arrowheads), reduced at 2 hphs (D), severely reduced at 4 hphs (E), and absent (asterisk) by 6 hphs (F). (G) Western blot analysis of anti-pSmad1/5/8 in control and dntcf3+ embryos. Protein was extracted at 2 hphs or 6 hphs and alpha tubulin was used as a loading control. (H) Histogram quantifying pSmad1/5/8 expression at 6 hphs, based on Western blots, in control and dntcf3+ embryos, levels normalized to alpha tubulin. (I) Histogram quantifying Edn1 expression by qPCR, in control and dntcf3+ embryos, p<0.01.

Because dntcf3+ embryos showed D-V defects in cartilage morphology and gene expression that more closely resembled Bmp- and Edn1-deficient embryos than dkk1+ we focused on dntcf3+. To examine interactions between Wnt and Bmp signaling in the arches we used an antibody that recognizes phosphorylated Smad1/5/8 (pSmad1/5/8) in dntcf3+ embryos. In controls pSmad1/5/8 localized to ventral arches 1 and 2 where levels of Bmp signaling have been shown to be highest at 24 hpf (Fig. 5A–C; [19]). Anti-pSmad1/5/8 staining was slightly reduced in the first arch at 2 hphs (24–26 hpf) in dntcf3+ embryos (Fig. 5D), in both arches by 4 hphs (Fig. 5E), and virtually lost altogether at 6 hphs (Fig. 5F). Western blots confirmed that pSmad1/5/8 levels were much lower than controls at 6 hphs (Fig. 5G, H). To examine potential interactions between Wnt and Edn1 signaling in the arches we performed qPCR for Edn1 in dntcf3+ embryos at 6 hphs. Edn1 expression was significantly reduced relative to control (Fig. 5I). These results reveal an indirect role for Wnts in D-V patterning through regulation of both Bmp and Edn1 signaling.

### Exogenous Bmp or Edn1 proteins partially rescue ventral patterning in Wnt-deficient arches

**Figure 6:**
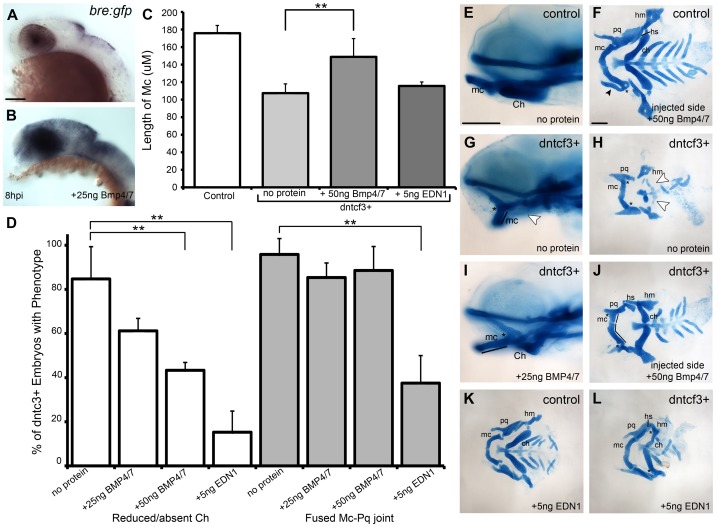
BMP protein rescues craniofacial phenotypes in dntcf+ embryos. (A,B) Whole mount ISH for *gfp* in *Tg(Bre:gfp)* embryos, lateral views, anterior to the left. (A) Wildtype and (B) following injection with 25 ng/embryo BMP4/7 into the arch region at 20 hpf. (C) Histogram quantifying Mc length (µM) in controls, dntcf3+ embryos, and dntcf3+ embryos injected with either 50 ng of BMP4/7 or 5 ng EDN1. (D) Histograms quantifying numbers of dntcf3+ embryos, alone or injected with BMP4/7 or EDN1 proteins, with reductions in Ch (left) and joint fusions between Mc and Pq (right). Numbers of embryos with rescue of Ch or Mc-Pq were averaged from three independent experiments (minimum of 10 embryos per protein injection experiment) and samples compared using a paired student t-test. (E, G, I) Whole mounted, alcian-stained 4 dpf larvae; control (E), dntcf3+ (G), and dntcf3+ injected with 25 ug BMP4/7 (I), lateral views, anterior to the left. Arrowhead in G indicates Ch loss. Black lines in G and I indicate Mc length and asterisks indicate Mc-Pq joint fusion. (F, H, J–L) Dissected, flat-mounted alcian-stained cartilages at 4 dpf, ventral views, anterior to the left; control with unilateral injection of 50 ng BMP4/7 (F), dntcf3+ (H), dntcf3+ with 25 ng BMP4/7 (J), control with 5 ng EDN1 (K) and dntcf3+ with 5 ng EDN1 (L). Arrowhead in F indicates duplicate Mc. Black lines in J indicate Mc length. Arrowheads in H indicate Sy and Ch. Asterisks in H, J and L indicate Mc-Pq joint fusions. **P<0.001. Abbreviations: Ch, ceratohyal; mc, Meckel's; mc', duplicated Meckel's; Sy, symplectic. Scale bar: 100 µm.

Bmps act together with Edn1 to promote ventral-intermediate cell fates in the arches [16], [17], [21], [22], [58]. Therefore we examined the ability of Bmp and Edn1 to restore ventral-intermediate gene expression in Wnt signaling-deficient embryos. Beads coated in human recombinant BMP4/7 heterodimers effectively induce Bmp target genes in zebrafish pharyngeal arches [19]. Similarly, microinjection of a 25 ng/nl BMP4/7 solution extracellularly on one side of the head induced Bmp signaling, as measured by expression of the transgenic Bmp-response element reporter (Bre:Gfp; [19]) at 8 hours post injection (hpi) (Fig. 6A, B). Unilateral injections of BMP4/7 protein into dntcf3+ embryos at 4 hphs partially rescued cartilage defects on the injected side (Fig. 6C–J). Typically this restored Mc length and Ch, but not the Mc-Pq joint, and rescue was dose-dependent (Fig. 6C, D). These results suggest that Wnt signaling acts upstream of, or possibly in parallel to, Bmp signaling to promote ventral cartilage cell fates in the arches. EDN1 protein injections have previously been shown to rescue an Edn1 mutant phenotype and partially rescue a Bmp loss of function phenotype [16], [19]. EDN1 injections into dntcf3+ embryos also partially rescued Mc length, but notably were more proficient at rescuing Ch and joint development (Fig. 6C–D, L).

**Figure 7:**
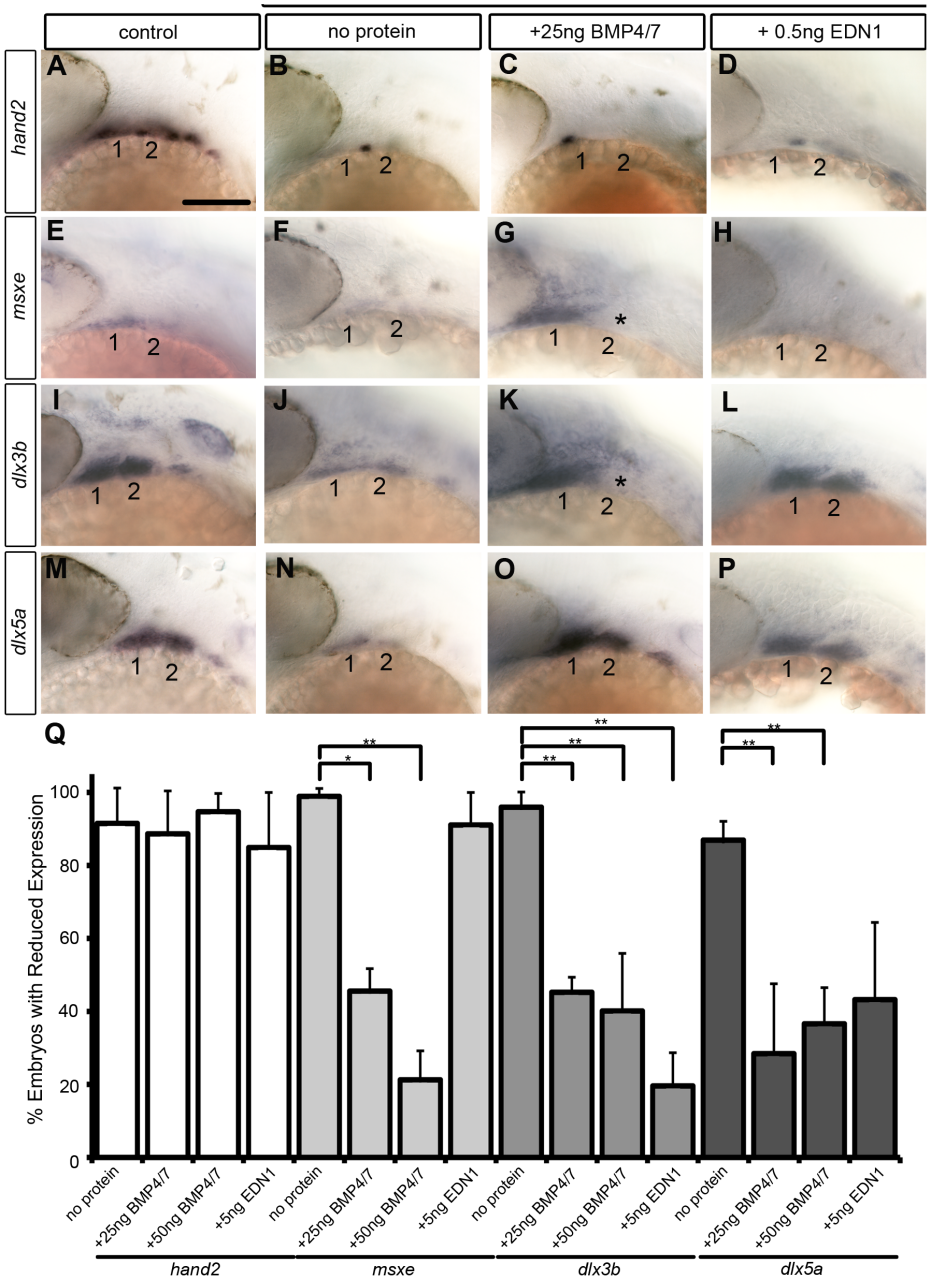
Bmp and Edn1 restore ventral-intermediate gene expression, but not *hand2*, in dntcf3+ embryos. (A–P) Whole mount ISH for ventral-intermediate patterning genes in heat shocked controls (A, E, I, M), dntcf3+ embryos (B, F, J, N), and dntcf3+ embryos with either 25 ng BMP4/7 (C, G, K, O), or 5 ng EDN1 (D, H, L, P), lateral views, anterior to the left. Asterisks in G and K indicate cases in which rescue occurred in arch 1 but not arch 2. (Q) Histogram quantifying the number of embryos with reductions in arch expression for each gene. Quantification obtained by counting number of embryos with rescue of gene expression in three independent experiments (minimum of 10 embryos per protein injection experiment). Statistical method used is a paired student t-test. * P<0.05, ** P<0.001. Scale bar: 100 µm.

While both Bmp and Edn1 signaling induce many of the same genes that specify ventral-intermediate NC cell fates in the early arches, by later stages Bmps become much stronger inducers of *hand2* (ventral) and *msxe* (ventral-intermediate) [19], [20]. Strikingly, neither BMP4/7 nor EDN1 protein injections at 4 hphs were sufficient to rescue *hand2* expression in dntcf3+ embryos (Fig. 7A–D, Q). BMP4/7 but not EDN1 restored *msxe* expression (Fig. 7E–H), particularly in the mandibular arch near the injection site (Fig. 7G). In contrast, both BMP4/7 and EDN1 injections restored *dlx3b* and *dlx5a* expression in the intermediate domain (Fig. 7I–P, Q). These results suggest that *hand2* expression absolutely requires Wnt signaling to respond to Bmps, while other signals can partially substitute for Wnts in induction of more intermediate-dorsal NC cell fates.

### Wnt signaling regulates expression of Bmp receptors

**Figure 8:**
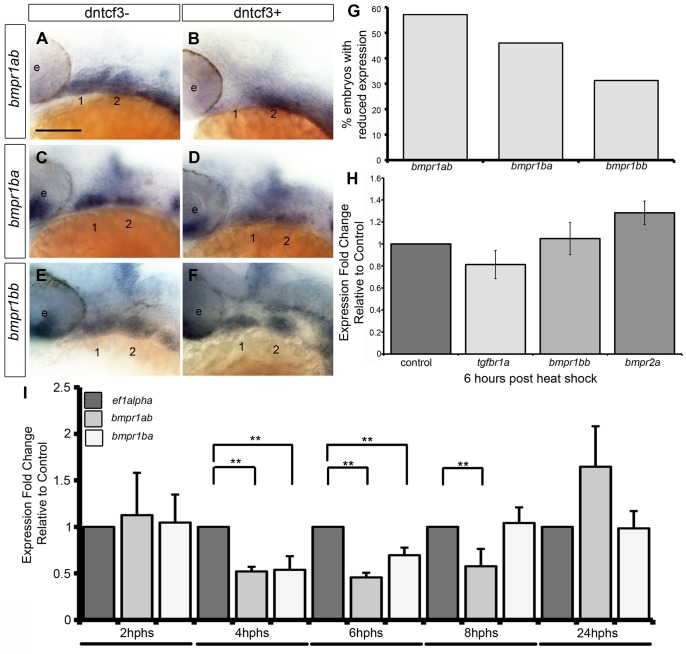
Bmp receptor expression in dntcf3+ embryos. (A–F) Whole mount ISH for *bmpr1ab* (A, B), *bmpr1ba* (C, D), and *bmpr1bb* (E, F) expression, lateral views, anterior to the left. (G) Histogram quantifying the number of dntcf3+ embryos with reduced expression. (H,I) qPCR analysis of Bmp receptor expression levels in dntcf3+ embryos at different times post heat shock, normalized to nontransgenic, heat-shocked controls, with *ef1alpha* as an internal control. * P<0.05, ** P<0.001. Abbreviations: e, eye; nc, neural crest. Scale bar: 100 µm.

To further investigate how Wnts might regulate the ability of NC cells to respond to Bmp signaling, we examined whether or not dntcf3+ embryos show any changes in expression of Bmp receptors. Zebrafish have four type 1 receptors (*Bmpr1aa*, *ab*, *ba*, *bb*) and two type II receptors (*Bmpr2a* and *b*). Whole mount ISH for all six receptors revealed that only *bmpr1ab*, *bmpr1ba*, and *bmpr1bb* are expressed strongly in the arches at 24 hpf (Fig. S9C–F, I–J, M–R). *bmpr1aa*, *bmpr2a*, and *bmpr2b* were detected much more broadly throughout the embryo at this stage (Fig. S9A–B, G–H, K–L). *Bmpr1ab* expression extended throughout arches 1 and 2, while *bmpr1ba* and *bmpr1bb* expression was restricted to more intermediate and ventral domains (Fig. S9M–O). Transverse sections additionally showed that *bmpr1ab* and *bmpr1ba* expression is limited to arch NC cells and not surrounding epithelia (Fig. S9P–R). In dntcf3+ embryos *bmpr1ab* was severely reduced (57% n = 35) (Fig. 8A–B, G), while *bmpr1ba* was slightly reduced (*bmpr1ab:* 46% n = 57) and *bmpr1bb* expression was largely unaffected (*bmpr1bb:* 31% n = 29) (Fig. 8C–F, G).

Changes in Bmp receptor expression in dntcf3+ embryos were further quantified by qPCR analysis. At 6 hphs we compared the relative expression of arch specific Bmp receptors (*bmpr1ab*, *bmpr1ba*, *bmpr1bb*) with ubiquitously expressed *bmpr2a* and *tgfbr1a*, a TGF-B receptor expressed in the arches unrelated to Bmp signaling [59]. There was no detectable reduction in *tgfbr1a* or *bmpr2a* expression in dntcf3+ embryos (Fig. 8H). At 6 hphs, both *bmpr1ab* and *bmpr1ba* expression were reduced (Fig. 8I) but *bmpr1bb* expression showed no difference from controls (Fig. 8H). A time series analysis revealed no change in *bmpr1ab* and *bmpr1ba* expression at 2 hphs, despite reduced Wnt signaling (see Fig. 1), but levels dropped dramatically by 4 hphs. *bmpr1ba* but not *bmpr1ab* expression recovered substantially by 8 hphs. This suggests differential requirements for Wnt signaling in induction of Bmp receptors.

### Dkk1b functions in the pharyngeal endoderm

dkk1+ embryos exhibit a unique clefting of the mandible not seen with dntcf3+. Although primarily known as a repressor of Wnt signaling, Dkk1 has also been reported to positively regulate the Wnt-PCP pathway [60]. To gain further insights into its tissue-specific functions, we examined *dkk1b* expression in pharyngeal arch primordia. Of the five known dkk genes in zebrafish, only *dkk1b* is expressed in the embryonic arches [61]. We found that between 28–48 hpf *dkk1b* expression localized to the pharyngeal endoderm, particularly the pouches between arches (Fig. S10A–C). Consistent with this, expression was lost in *van gogh* (*vgo*) mutants, which lack pouches [10] (Fig. S10D–E). *dkk1b* expression was also detected in the stomodeum (oral ectoderm) at 28 hpf (Fig. S10A) and later in the ectoderm of the mouth at 48 hpf (Fig. S10F).

Signals from the pharyngeal endoderm and oral ectoderm are necessary for craniofacial patterning and chondrogenesis [9], [10], [13]–[15]. To determine if there were gross defects in these epithelial layers in dntcf embryos, we examined *nkx2.3*, and found that its expression in the pharyngeal endoderm was disorganized in the first two pouches (Fig. S11B,F) and severely reduced in the more posterior pouches (Fig. S11B). In contrast, anterior pouches appeared unaffected in dkk1+ embryos, while the more posterior pouches were occasionally disorganized (Fig. S11D,H). Expression of *pitx2ca* in the oral ectoderm (Fig. S11I–R) was delayed in dkk1+ embryos until 26 hphs (Fig. S11O), by 30 hphs the mouth opening was abnormally elongated laterally and by 51 hphs showed a ventral midline fold (Fig.S11P–R). Thus pharyngeal pouch and mouth defects differ between dntcf and dkk1 embryos, which could account for some of the differences in their effects on growth and morphogenesis of the lower jaw.

## Discussion

**Figure 9:**
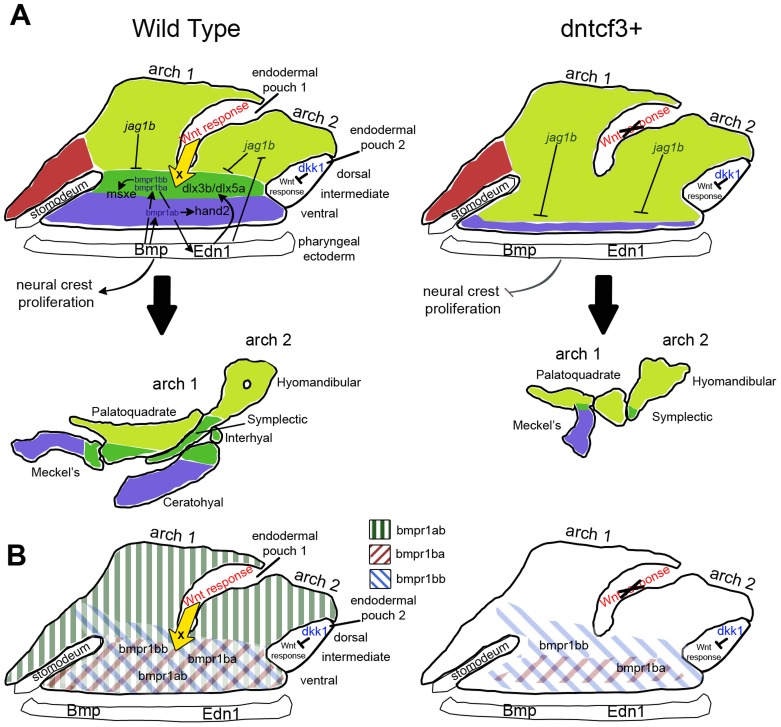
Model for the role of Wnt in dorsal-ventral arch patterning. (A) Diagrams illustrating regulation of expression of dorsal-ventral (D-V) arch patterning genes in the skeletogenic neural crest at 24 hpf (colored regions) by Bmp and Edn1 signals from the pharyngeal ectoderm and Wnt responses in the pharyngeal endoderm. Each arch is subdivided into ventral (blue), intermediate (dark green), and dorsal (light green) domains, which correspond to eventual dorsal-intermediate-ventral cartilage identities. Arrows indicate inductive influences by each signal on target genes. The yellow arrow indicates an unknown ventralizing signal X from the endoderm induced in response to Wnt. (B) Diagrams illustrating regulation of Bmp receptor expression along the D-V axis at 24 hpf (colored diagonal bars) by Wnt, Bmp and Edn1.

We show that Wnt signaling promotes proliferation and provides ventral-intermediate patterning cues to NC cells in the pharyngeal arches by participating in a regulatory network with Edn1 and Bmp (Fig. 9). By overexpressing Dkk1 or dnTcf3 to disrupt Wnt signaling, we show that Wnt promotes expression of ventral (*hand2*) and ventral-intermediate genes (*dlx3b*, *dlx5a*, *msxe*) and their corresponding skeletal derivatives, and acts upstream or in parallel to the ventralizing activities of Edn1 and Bmp. Unlike Edn1 and Bmp, however, our chimeric analyses suggest that direct responses to Wnt signaling occur in the pharyngeal endoderm, which also expresses *dkk1*. This endoderm must secondarily produce as yet unknown signals important for D-V patterning, which regulate the competence of NC cells to respond to Bmp signaling, in part by transcriptionally regulating Bmp receptors. Overexpression of dkk1 also causes a unique midline clefting of the mandible, which we suggest reflects a role in formation of the mouth.

### Wnt-mediated patterning and growth in craniofacial development

Direct Wnt responses in the ventral first and second arches resemble the pattern of TOP:Gal expression in mice, including distal (ventral) arch 1 [36], [37]. *Mycn*, a direct transcriptional Wnt target, is also expressed in both fish and mouse arch NC cells, where it is likely to inhibit Wnt-β-catenin signaling [62], and provide negative feedback. Murine *Mycn* is expressed in highly proliferative cells and mutants show hypoplasia of the mandibular arch [63], [64]. Similarly, we find reduced proliferation in the pharyngeal arches in Wnt-deficient zebrafish and smaller craniofacial cartilages (Fig. 3; Fig. S5). Thus, Wnt signaling may promote growth of the ventral arches through induction of *mycn* expression.

We show a critical requirement for Wnt signaling in arch growth and patterning that is distinct from its earlier roles in NC induction and migration. Earlier heat shocks of dntcf3 or dkk1 zebrafish (10–20 hpf) disrupt premigratory NC formation [41], [65] similar to *Wnt1*/*Wnt3a* mutant mice [43]. Unlike recent conditional loss- and gain-of-function studies of βcat in the pharyngeal ectoderm in mice [44], we find no changes in cell survival in the arches in dntcf3+ embryos.

D-V defects in gene expression in the arches caused by overexpressing dntcf3 or dkk1 at these later stages point to a problem with canonical Wnt signaling. Both reduce expression of canonical Wnt target genes up to 8 hphs (Fig. 1I; Fig. S1), and both lead to ventral cartilage and joint defects. However, dkk1 overexpression has more subtle effects (restricted primarily to Mc and the jaw joint) than dntcf3. Defects in dkk1+ embryos are also stronger when heat shocked at slightly earlier stages (15–22 hpf), than dntcf3+ (22–24 hpf). These differences may reflect distinct functions for the two transgenes, or a delay due to the time required for Dkk1 to competitively bind with the Lrp5/6 co-receptor, whereas Tcf3 directly binds βcat and Wnt target genes. Heat shocking dntcf3 or dkk1 at earlier stages eliminates cartilage, consistent with requirements for canonical Wnt signaling in NC induction. Both *mycn* and *axin2* expression recover by 24 hphs in heat shocked embryos indicating a transient requirement for canonical Wnt signaling prior to skeletal cell differentiation.

### Wnt and the D-V signaling network in pharyngeal arch development

Bmps and Edn1 secreted by the pharyngeal ectoderm both promote ventral-intermediate skeletal cell fates in the arches [19], [20] and our results implicate Wnt as an additional ventralizing factor. Overexpression of dntcf3 leads to reduced *hand2*, *dlx3b*, and *dlx5a* ventrally and expansion of dorsal *jag1b* expression (Fig. 4), and similar but less severe changes in gene expression result from dkk1 overexpression. Loss of the positive Wnt regulator, R-spondin, in mice also disrupts expression of *Hand2*, *Dlx5*, *Dlx6*, and *Msxe* [66], suggesting a conserved requirement for Wnt signaling in promoting ventral-intermediate cell fates.

How are these different ventralizing signals integrated during D-V arch patterning? Wnts can either activate or inhibit Bmp signaling in different developmental contexts [67]–[71]. pSmad1/5/8 expression is reduced in the pharyngeal arches of dntcf3+ embryos (Fig. 5), suggesting a novel role for Wnts upstream of Bmp signaling during arch development. Consistent with this model, microinjection of Bmp4/7 protein directly into the arch primordia at 4 hphs rescues ventral cartilages (Mc, Ch) in dntcf3+ embryos, but not joint fusions (Fig. 6C, F). Similar injections of BMP4/7 protein into *edn1* ^−/−^ mutant zebrafish rescues ventral cartilages but not joint fusions, while ectopic Edn1 can rescue joint defects caused by a loss of Bmp [19]. We show that injection of EDN1 protein rescues joint fusions in Wnt deficient embryos (Fig. 6D,L). Wnts also induce Edn1 expression in the pharyngeal ectoderm [66]. Taken together, these results suggest that Wnt signaling influences ventral cell fates in the arches through both Bmps and Edn1, and joint patterning specifically through Edn1.

Another clue to specificity in the D-V arch patterning system comes from the fact that *msxe* expression (a direct BMP target and marker of more intermediate identities) is induced by Bmp4/7 in *edn1* ^−/−^ mutants [20], and by Edn1 protein in the absence of Bmp signaling [19]. Surprisingly, however, *msxe* expression in dntcf3+ embryos is only rescued by BMP4/7, and not EDN1, while *dlx3b* and *dlx5a* expression is rescued in both. This suggests that Edn1 can only induce *msxe* expression in the arches in the presence of Wnt signaling. Thus Wnt controls the competence for arch cells to respond to Edn1 in addition to inducing expression of Edn1 itself. Mice mutant in the essential Wnt receptor co-factor, Lrp6, also lack expression of M*sx1* and *Msx2*, in the arches [72], possibly as a result of defects in Bmp signaling.

Neither BMP4/7 nor EDN1 overexpression rescues *hand2* expression in dntcf3+ embryos, revealing a critical requirement for Wnt in induction of *hand2* [17], [23], [73]–[75]. Both Bmp and Edn1 induce *hand2* expression and specify the ventral arch domain initially, but later Bmps maintain *hand2* in the ventral domain while *Edn1* promotes expression of more ventral-intermediate genes. We pinpoint a critical period for Wnt signaling in D-V patterning between 24–30 hpf, when *hand2* expression is unresponsive to Edn1. Our results suggest that Bmp signaling requires Wnt signaling to induce *hand2* expression in the arches. Consistent with this model, Wnt signaling directly regulates Hand2 transcription in chondrocytes [76]. Failure of *hand2* induction is not simply due to loss of cells, since ventral NC cells are still present in the arches of dntcf3+ embryos (Fig. 4N–O; Fig. S7). BMP protein can also induce *hand2* throughout the D-V extent of the arch [20]. Our results suggest that Wnt signaling plays a critical role in regulating the competence of cells to respond to BMPs and to express *hand2*.

We propose that Wnt signaling activates a signal (factor X) from the pharyngeal endoderm that primes the ability of NC cells to respond to Bmp signaling, in part through the transcriptional regulation of Bmp receptors (Fig. 9). Similarly in D-V patterning of the mouse limb Wnt signaling is thought to act upstream of Bmpr1a [71]. Three type I Bmp receptors, *bmpr1ab*, *bmpr1ba*, *bmpr1bb*, have arch-specific expression in zebrafish, similar to mice [77]. These are expressed in nested patterns within the arches: *bmpr1ab* throughout and *bmpr1ba/bb* only in intermediate-ventral domains. Thus Bmpr1 receptors may have distinct roles in different spatial domains (in addition to their cell-type specific roles [78], [79]), but this has been difficult to test due to early lethality in traditional *Bmpr* knockouts [80], [81]. We show that overexpression of dntcf3+ inhibits *bmpr1ab* and *bmpr1ba*, but not *bmpr1bb* and *bmpr2a*, expression in the arches. This reduction in Bmpr expression occurs later than most direct Wnt targets (Fig. 8I; 4–8 hphs) suggesting that it is indirect, consistent with our model that Wnt activates an unknown signal from the endoderm important in this process. *bmpr1ba* expression also recovers by 8 hphs, before *bmpr1ab* expression and within the period during which Wnt signaling is significantly reduced (Fig. 1I) indicating that *bmpr1ab* is particularly sensitive. This could help explain the inability of Bmp protein to rescue *hand2* expression in dntcf3+ embryos if *bmpr1ab* plays a specific role in *hand2* induction. In contrast, intermediate-ventral genes such as *msxe* and *dlx3b*, may be rescued by Bmp protein because other Bmp receptors are sufficient for their induction. Such distinct transcriptional roles for Bmp receptors could help fine-tune D-V domains within an arch despite the relatively broad expression of Bmp ligands.

### Distinct roles for Wnt signaling in pharyngeal endodermal development

Both dntcf3+ and dkk1+ embryos appear to function in the pharyngeal endoderm, which is an important signaling center in craniofacial development [7], [9]. Our chimeric analyses demonstrate a cell autonomous requirement for dntcf3 in endoderm (Fig. S2) and *dkk1* expression is restricted to this epithelium. Interestingly, we do not detect *dkk1* expression in the first pharyngeal pouch, which lies between arches 1 and 2 (Fig. S10). This could help explain why Wnt signaling only appears to be required for D-V patterning in these arches; the more posterior ceratobranchials (arches 3–7) are largely unaffected in heterozygous dkk1+ and dntcf3+ embryos (Fig. 2B,F). These results suggest a previously unrecognized role for Wnts and Wnt antagonists in endoderm and the existence of an as yet unknown factor X produced by endoderm that is important in D-V patterning of the NC (see Fig. 9).

The distinct and highly penetrant clefting of the mandible observed in dkk1+ embryos is never observed in dntcf3+ embryos. Disruption of the canonical Wnt pathway can cause clefting of the palate in mice [36], [46], [67], but such midline clefts in the lower jaw are rare. Midline facial defects, particularly of the frontonasal process, have been reported in Wnt signaling mutants in mice [36], [66], [67]. Mice mutant for Dlx5/6 have cleft mandibles and Wnt9b mutants have cleft lip [82], [83]. Humans with Richieri-Costa-Perieira syndrome also exhibit clefting of the lower jaw similar to what we describe in dkk1+ embryos [68], [84]. Dkk1 not only inhibits canonical Wnt signaling [52] but can also activate the non-canonical Wnt-PCP pathway during zebrafish gastrulation [60]. Non-canonical Wnt signaling has also been implicated in craniofacial midline development as Wnt5a mutant mice have clefting of the secondary palate [66]. Thus, overexpression of Dkk1 may lead to both a canonical Wnt/βcat loss-of-function and a non-canonical Wnt-PCP gain-of-function to cause lower jaw clefting.

Overexpression of dkk1 also leads to elongation and ventral clefting of the mouth, which is not observed in dntcf3+ embryos. Both loss- and gain-of-function mutations in mammalian Dkk1 result in midline clefts in the frontonasal and maxillary prominences [36]. NC cells fated to form the lower jaw lie adjacent to the oral ectoderm (stomodeum), which secretes important skeletogenic signals such as Shh [14], [15] and Bmps [19]. *dkk1b* transcripts are normally restricted to the anterior ectoderm of the mouth opening (Fig. S10F) where both *fgf8* (distal) and *shh* (medial) are expressed. Thus, misexpressing *dkk1b* throughout the oral ectoderm may disrupt one of these other signals. Future experiments are needed to determine the roles of Wnt signaling in mouth development and the causes of mandibular clefts.

## Materials and Methods

### Ethics statement

All zebrafish work was performed using protocols approved by the University of California, Irvine Institutional Animal Care and Use Committee (Protocol # 2000-2149-4).

### Animals

Adult *Tg(hsp701:dkk1-GFP)* (dkk1) [50] and *Tg(hsp70I:tcf3-GFP)* (dntcf3) [41] fish were genotyped by performing PCR analysis for *gfp* (sense, GTGATGCAACATACGGAAAAC; antisense, GCCATGTGTAATCCCAGCAGC) using genomic DNA extracted from fin clips as template. Zygosity of fish was determined by outcrossing genotyped transgenic adults with wild type adults and scoring the Mendelian ratio of GFP positive to GFP negative after heat shocking as described below. To observe NC, we outcrossed adult homozygous *Tg(hsp70I:tcf3-GFP)* fish with sox10;LynTdtomato fish to make a stable double transgenic line. Adult heterozygous *Tg(7xTCF-Xla.Siam:GFP)^ia4^* (7xTCF;GFP) transgenics [49] were in-crossed and sorted by GFP expression for downstream phenotypic analysis. *Tg(BRE:gfp)* (bre:gfp) [19] heterozygous adults were in-crossed, selected for strong GFP expression, and used for protein injection (see below).

### Heat shock conditions

Heat shocks were performed in a thermal cycler at 39°C for either 12 min (dntcf3) or 30 min (dkk1). Fluorescence was checked 1-hour post heat shock and GFP-negative embryos were separated and used as controls. Embryos were then raised in a 28.8°C incubator until they were fixed for *in situ* analysis, RNA extraction, or protein extraction at various time points after heat shock or 4 dpf for skeletal staining.

### Phenotypic analysis

Alcian blue staining of cartilage was performed on 96 hpf embryos as previously described [19]. *In situ* hybridization was performed on embryos fixed in 4% PFA for one hour at room temperature. Probes used include *gfp* [19], *dlx2a*, *dlx3b*, *dlx6a* [85], *mycn* [86], *hand2* [87], *jag1b* [28], *msxe*, *dkk1b*, *nkx2.3* [17], *ednrA1*, *ednrA2* (Nair et al, 2007), and *pitx2ca* [88]. The *axin2a* probe was synthesized directly from a PCR product with a T7 promoter site added at the 3′ end (sense, AGAAGATGACCCACGTCCAC; antisense, TAATACGACTCACTATAGGGGACTGTGACCTTGTGCTGAGAC). The Bmp receptor probes were synthesized directly from PCR products with a T7 promoter site added at the 3′ end: Bmpr1aa (sense, TAGCCAACCCCAATGCTTAC; antisense, TAATACGACTCACTATAGGGCCCATTTGTCTCGCAGGTAT), Bmpr1ab (sense, GATGCCACAAACAACACCTG; antisense, TAATACGACTCACTATAGGGACTTTCACCGCCACATTTTC), Bmpr1ba (sense, AGAATCTCTGCGGGATCTCA; antisense, TAATACGACTCACTATAGGGGCTCCGTTTCTCTTGACCAG), Bmpr1bb (sense, TCACGGATTATCACGAGAGCG; antisense, TAATACGACTCACTATAGGGATTATGAGCCCAGCACTCGC), Bmpr2a (sense, CCACAATGACACCTCAGTGG; antisense, TAATACGACTCACTATAGGGTTAGGGACGTTCTGCTGCTT), Bmpr2b (sense, TATTGTCGCGCTGTTCTTTG; antisense, TAATACGACTCACTATAGGGGCAGATAGGCCAGTCCTCTG). A *pcna* probe was generated by a T7 promoter from a clone of the ORF (Open Biosystems, clone ID:7000501) in pExpress1 after linearization with *EcoRI*. Immunolabeling was performed using 1∶500 rabbit anti-phosphoHistodone3 (Upstate Biotechnology), 1∶1000 rabbit anti-pSmad1/5/8 (Millipore), or 1∶500 chick anti-gfp (AbCam) antibodies diluted in 1% DMSO, 0.5% Triton ×100 in PBS and detected using 1∶1000 dylight donkey anti-rabbit 564 (Jackson ImmunoResearch Laboratories) or 1∶1000 donkey anti-chick dylight 488 (Jackson ImmunoResearch Laboratories).

### Protein injection

*Tg(hsp70I:tcf3-GFP)* embryos were heat shocked as described, anesthetized, and then embedded in 1% low melt agarose in embryo medium. Human EDN1 (Sigma-Aldrich) was diluted to 10 µg/µl and human recombinant BMP4/7 (R&D Systems) was diluted to either 50 ng/nl or 10 ng/nl. A 0.5 nl droplet of protein solution was pressure injected into the arch region 4 hours post heat shock (∼26 hpf) using a glass needle. Embryos were then carefully removed from the agarose using forceps and fixed for phenotypic analysis 4 hours later (∼30 hpf) or 4 dpf for alcian stain using 4% PFA at room temperature for 1 hour.

### 6-bromoindirubin-3′-oxime (BIO) treatment

Dechorionated control, dkk1+, and dntcf3+ embryos were placed in dishes containing 50 µm or 100 µm of BIO (30 mm stock in DMSO) (Sigma) diluted in embryo medium (EM) at 2 hours post heat shock [55]. The dishes were placed in a 28.8° incubator for 6 hours and then washed several times with EM. Embryos were fixed for in situ hybridization, harvested for RNA, or allowed to develop to 4 dpf and then fixed for alcian blue staining.

### Western blot

Protein was extracted from dechorionated embryos by adding 2 µl/embryo of sample buffer (60 mM Tris-HCl pH 6.8, 2% SDS, 10% glycerol, 5% β-mercaptoethanol, 0.01% bromophenol blue) and homogenizing with a pestle. The sample was boiled in a 95°C heat block for 10 min and then immediately placed on ice. Before loading into a 10% SDS gel the sample was spun down at 1300 rpm for 5 min. The membrane was blocked in 3% BSA and 3% Donkey Serum in TBST (1XTBS −20 mM Tris-HCl, 150 mM NaCl, 0.1% Tween) for one hour at room temperature and incubated overnight at 4°C with 1∶1000 rabbit anti-pSmad1/5/8 (Cell Signaling). The next day the membrane was washed several times in TBST and then incubated with 1∶5000 donkey anti-rabbit HRP (GeneTex) for 1 hour at room temperature.

### Quantitative real time PCR analysis

Total RNA was extracted from control, dkk1+, and dntcf3+ embryos at various time points past heat shock using Trizol reagent (Ambion). cDNA synthesis was performed with 1 µg of RNA using Protoscript M-MuLV First Strand cDNA synthesis kit (New England Biosystems). qPCR was performed using Light Cycler 480 SYBR Green Master (Roche Applied Science) in a Light Cycler 480 Real Time PCR machine. Q-PCR primer sets used were for *mycn* (Sense-AACAAGAGGGAGAATGCCA; Antisense-TAGAAGTCATCCTCGTCCG), *axin2* (Sense- CAATGGACGAAAGGAAAGATCC; Antisense-AGAAGTACGTGACTACCGTC), *lef1* (Sense-CCAGACATTCCCAATTTCTATCC; Antisense-GTGATGTGAGAACCAACCC), *ef1alpha* (Sense- CAAGGGATGGAAGATTGAGC; Antisense- AACCATACCAGGCTTGAGGA), *bmpr1ab* (Sense- CATGAGGGAAGTGGTATGTG; Antisense- ATGACTCGTAAGCACTCGT), and *bmpr1ba* (Sense- GACAATATACTGGGATTTATAGCGG; Antisense – ATGATAGTCTGTGATCAGGTAGAG), *bmpr1bb* (Sense-AACATACTGGGCTTCATCG; Antisense- CTCGTGATAATCCGTGATCAG), *bmpr2a* (Sense-TTTCCCAGGTGAAACAGTG; Antisense-TGCATGTCCTCTATGGTAGG), *tgfbr1a* (Sense-GCATGATCAAGCTGTCTCTG; Antisense-CAGGCTTACCCTGAGTACC), and *edn1* (Sense-TATGGGTGAACACACCAGAGCGAA; Antisense-CGCTTGGCAGAATGAAGAGCATGT).

### Chimeric analyses

*Tg(hsp70I:tcf3-GFP)* donor embryos were injected at the 1-cell stage with 15 pg Taram-A (Tar*) mRNA, which drives cells to an endodermal fate, combined with a 1∶1 mixture of 6% biotin-dextran and 6% rhodamine-dextran. Cells were transplanted into WT hosts at the 30–50% epiboly stage (5 hpf) to generate chimeras, as described previously [9]. Host embryos with red fluorescent cells in the pharyngeal endoderm were sorted at 22 hpf, heat-shocked and raised to either 30 hpf for ISH or 5 dpf for skeletal staining. Grafts were labeled at either time-point using a peroxidase-coupled streptavidin and diaminobenzidine.

## Supporting Information

Figure S1 Wnt signaling defects in dkk1+ embryos. (A–D) Whole mount in situ hybridization (ISH) for *mycn* (A, B) and *axin2* (C, D) expression in dkk1+ embryos at 24 hpf (2 hphs), lateral views, anterior to the left. Arrowheads indicate expression at the mid-hindbrain boundary. (E) Quantitative, real-time PCR (pPCR) analysis for *axin2* (red bars) and *lef1* (orange bars) in dkk1+ embryos, normalized to nontransgenic, heat-shocked controls, with *ef1alpha* as an internal control. Abbreviations: e, eye.
(TIF)

Figure S2 Wnt signaling in the pharyngeal endoderm is necessary for D-V arch patterning. (A) Dissected, flat mounted alcian blue/alizarin stained cartilage/bone at 5 dpf. Anterior to the left. Transplantation of dntcf3+ endoderm results in D-V patterning defects including reduced Mc, fused Mc-Pq, and loss of Hs. A dotted line indicates the extent of the endoderm transplant, which was lost in dissection. Asterisk indicates joint fusion. (B–C) Lateral views of *in situ* hybridization for *jag1b* in a control (B) and a mosaic embryo in which dntcf3+ cells were transplanted into the endoderm in WT hosts. White arrowheads indicate expansion of *jag1b* expression into the ventral domain of arches 1 and 2. Asterisks indicate transplanted, diaminobenzidine-stained donor cells. Abbreviations: Ch, ceratohyal; Hm, hyomandibular; Hs, hyosymplectic; Mc, Meckel's; Pq, palatoquadrate.
(TIF)

Figure S3 BIO rescues expression of Wnt targets in dntcf3+ and dkk1+ embryos. (A–F) Whole mount ISH for *gfp* (A–C) and *axin2* (D–F) at 30 hpf in embryos treated with DMSO, 50 µM BIO, and 100 µM BIO for 6 hours at 24 hpf. With increasing concentrations of BIO, 7xTCF:GFP embryos show an increase in ectopic *gfp* expression, particularly throughout the brain (B,C), Similarly, *axin2* expression expands in a dose-dependent manner (E,F). (G–H) qPCR analysis for Wnt targets *lef1* (G) and *axin2* (H) in control, dntcf3+, and dkk1+ embryos treated with DMSO, 50 µM BIO, and 100 µM BIO for 6 hours at 2 hphs. Red line indicates normalized expression levels in DMSO treated non-transgenic controls with *ef1alpha* as an internal control. BIO induces expression of Wnt targets in non-transgenic controls and rescues expression in dntcf3+ and dkk1+ embryos (G,H).
(TIF)

Figure S4 BIO rescues D-V defects in dntcf3+ and dkk1+ embryos. (A–I) Dissected, flat mounted alcian-stained cartilage at 4 dpf, ventral views, anterior to the left; non-transgenic control (top row), dntcf3+ (middle row) and dkk1+ (bottom row), untreated (A, D, G), or treated with 50 µM (B, E, H) or 100 µM (C, F, I) BIO for 6 hours. White arrowheads in G-H indicate Mc clefting, and black arrowheads indicate missing Ch in D. Asterisks in D and G indicate Mc-Pq joint fusions. High concentrations of BIO reduce overall cartilage size and cause specific loss of dorsal cartilages (C). BIO treatments rescue Mc-Pq joint fusions in both dntcf3+ and dkk1+ embryos (E, F, H, I, M) as well as Ch in dntcf3+ embryos (E, F, M). High BIO concentrations rescue Mc clefting in dkk1+ embryos (I). (J–L) Whole mount ISH for *pcna* in control embryos treated with DMSO, 50 µM BIO, and 100 µM BIO. (M) Histogram quantifying the percentage of dntcf3+ embryos showing Ch loss or Mc-Pq joint fusion in response to BIO treatments. Abbreviations: Ch, ceratohyal; Hm, hyomandibular; Mc, Meckel's; Pq, palatoquadrate.
(TIF)

Figure S5 Cell death is not increased in dkk1+ and dntcf3+ embryos. (A–H) Lateral views of live acridine orange staining at 6 hphs in control (A, B, E, F), dkk1+ (C, D), and dntcf3+ (G, H) embryos. White arrowheads indicate apoptotic cells. Abbreviations: e, eye.
(TIF)

Figure S6 Arch proliferation defects in dkk1+ and dntcf3+ embryos. (A, B, D, E) Anti-phosphoHistone3 (pH 3) staining in controls (A, A′) and dkk1+ (B, B′) embryos stained 3 hphs and in controls (D, D′) and dntcf3+ (E, E′) embryos stained 4 hphs; lateral views, anterior to the left. (A′, B′, D′, E′). Enlargements of boxed areas encompassing presumptive first and second arches used for quantification. (C, F) Histograms quantifying numbers of pH 3 positive cells in the pharyngeal region of control and dkk1+ embryos, p<0.05 (C) and dntcf3+ embryos, p = 0.06 (F). Scale bar: 100 µm.
(TIF)

Figure S7 Neural crest migration in dntcf3+ embryos. (A–H) Live images of *Tg(sox10:lyn-Tdtomato)* fluorescence in cranial neural crest cells in the arches in control (A–D) and dntcf3+ (E–H) embryos at 2 hour intervals post heatshock, lateral views, anterior to the left.
(TIF)

Figure S8 BIO disrupts dorsal-ventral patterning gene expression. (A–L) Lateral views of whole mount ISH for D-V patterning genes in embryos treated with DMSO (A, D, G, J), 50 µM BIO (B, E, H, K), and 100 µM BIO (C, F, I, L) for 6 hours at 24 hpf. With increasing concentrations of BIO, expression of the ventral-intermediate gene *msxe* expands dorsally (B, C) (black arrowheads). In response to BIO treatments, expression domains of *hand2* (H–I) and *dlx3b* (E–F) only expand slightly. Expression of the dorsally restricted *jag1b* is reduced (asterisk) in the first and second arches of BIO treated embryos (K–L).
(TIF)

Figure S9 Bmp receptor expression during pharyngeal arch development. (A–O) Whole mount ISH for *bmpr1aa* (A, B), *bmpr1bb* (C, D, O), *bmpr1ab* (E, F, M), *bmpr2a* (G, H), *bmpr1ba* (I, J, N), and *bmpr2b* (K, L), lateral views, anterior to the left. (P–R) Transverse sections through 24 hpf embryos showing expression of *bmpr1ab* (P), *bmpr1ba* (Q), and *bmpr1bb* (R) in neural crest cells of the pharyngeal arches. Arrowheads indicate ventral restriction of *bmpr1ab* (P) and *bmpr1bb* (Q) expression compared with *bmpr1ab* (R). Abbreviations: e, eye; hb, hindbrain; mhb, mid-hindbrain boundary; nc, notocord; nt, neural tube; pa, pharyngeal arches; s, somites.
(TIF)

Figure S10 Dkk1 expression in pharyngeal endoderm. (A–F) Whole mount ISH for *dkk1b* in wild type (A–D, F) and *van gogh (vgo)* (E) embryos, (A–E) lateral and (F) ventral views, anterior to the left. Arrowhead in A indicates expression in the first arch. Asterisks in A and B indicate the first pharyngeal pouch. Arrowheads in C-E indicate the first and second pouches. (F) Ventral view at 48 hpf showing *dkk1b* expression in the oral ectoderm (arrowhead) surrounding the mouth opening (dotted line). Scale bar: 100 µm.
(TIF)

Figure S11 Defects in endoderm and oral ectoderm in dkk1+ embryos (A–L, N–Q) Whole mount ISH, anterior to the left. (A–H) Dorsal and lateral views showing *nkx2.3* expression in pharyngeal pouch endoderm at 50 hpf in controls (A, C, E, G), dntcf3+ (B, F), and dkk1+ (D, H) embryos. Normal pouches are numbered. Asterisks and black lines indicate defective pouches. (I–L, N–Q) Ventral views showing *pitx2ca* expression in control (I–L) and dkk1+ (N–Q) embryos. (M, R) Transverse sections of the mouth showing *pitx2ca* expression in control (M) and dkk1+ (R) embryos at 51 hpf. Arrowheads indicate expression in oral ectoderm. Dotted lines in K, L, P and Q indicate the mouth opening. White arrowhead indicates a ventral midline fold in the mouth. Scale bars: 100 µm.
(TIF)
