# Supplementary figures and images for: Wnt Signaling Interacts with Bmp and Edn1 to Regulate Dorsal-Ventral Patterning and Growth of the Craniofacial Skeleton
Source: PLoS Genet. 2014 Jul 24;10(7):e1004479. doi: 10.1371/journal.pgen.1004479 (PMC4109847; doi:10.1371/journal.pgen.1004479)

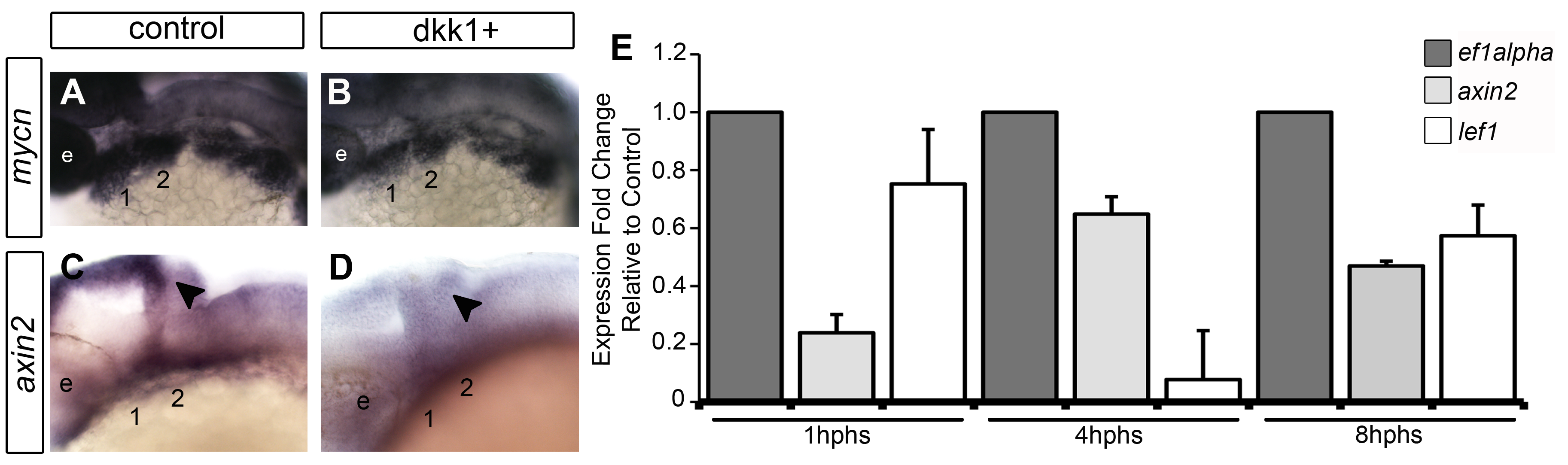

Supplement: Figure S1 — Wnt signaling defects in dkk1+ embryos. (A–D) Whole mount in situ hybridization (ISH) for mycn (A, B) and axin2 (C, D) expression in dkk1+ embryos at 24 hpf (2 hphs), lateral views, anterior to the left. Arrowheads indicate expression at the mid-hindbrain boundary. (E) Quantitative, real-time PCR (pPCR) analysis for axin2 (red bars) and lef1 (orange bars) in dkk1+ embryos, normalized to nontransgenic, heat-shocked controls, with ef1alpha as an internal control. Abbreviations: e, eye. (TIF) [file pgen.1004479.s001.tif]

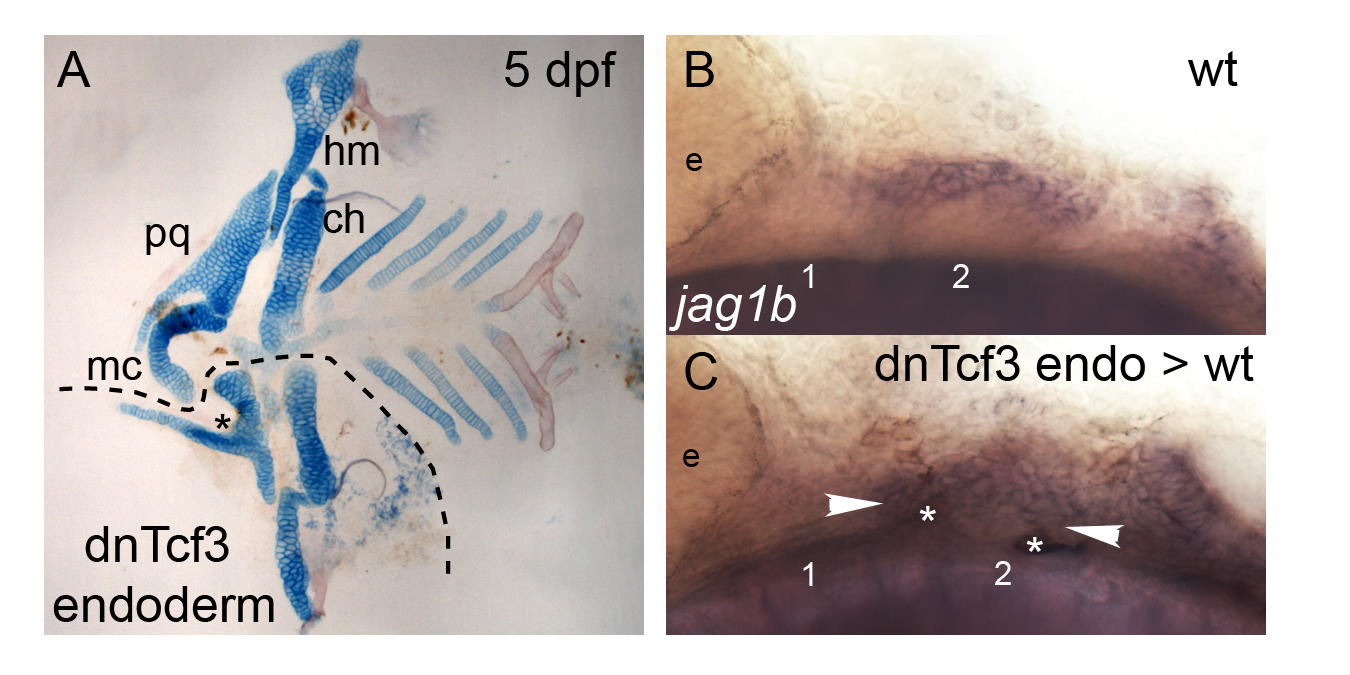

Supplement: Figure S2 — Wnt signaling in the pharyngeal endoderm is necessary for D-V arch patterning. (A) Dissected, flat mounted alcian blue/alizarin stained cartilage/bone at 5 dpf. Anterior to the left. Transplantation of dntcf3+ endoderm results in D-V patterning defects including reduced Mc, fused Mc-Pq, and loss of Hs. A dotted line indicates the extent of the endoderm transplant, which was lost in dissection. Asterisk indicates joint fusion. (B–C) Lateral views of in situ hybridization for jag1b in a control (B) and a mosaic embryo in which dntcf3+ cells were transplanted into the endoderm in WT hosts. White arrowheads indicate expansion of jag1b expression into the ventral domain of arches 1 and 2. Asterisks indicate transplanted, diaminobenzidine-stained donor cells. Abbreviations: Ch, ceratohyal; Hm, hyomandibular; Hs, hyosymplectic; Mc, Meckel's; Pq, palatoquadrate. (TIF) [file pgen.1004479.s002.tif]

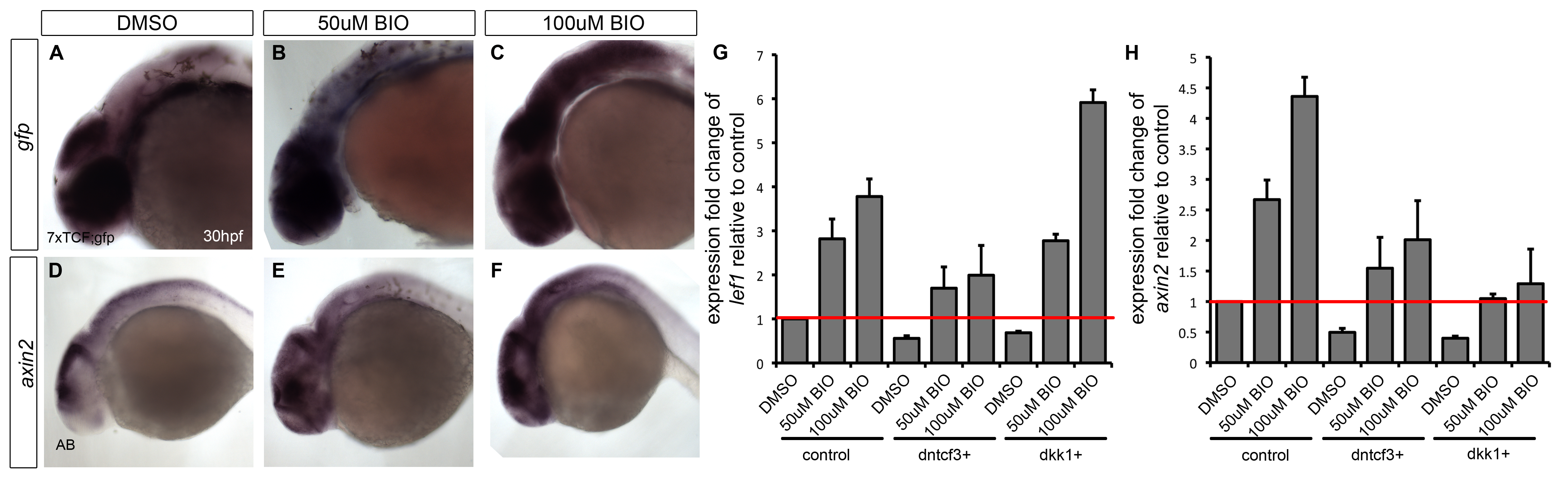

Supplement: Figure S3 — BIO rescues expression of Wnt targets in dntcf3+ and dkk1+ embryos. (A–F) Whole mount ISH for gfp (A–C) and axin2 (D–F) at 30 hpf in embryos treated with DMSO, 50 µM BIO, and 100 µM BIO for 6 hours at 24 hpf. With increasing concentrations of BIO, 7xTCF:GFP embryos show an increase in ectopic gfp expression, particularly throughout the brain (B,C), Similarly, axin2 expression expands in a dose-dependent manner (E,F). (G–H) qPCR analysis for Wnt targets lef1 (G) and axin2 (H) in control, dntcf3+, and dkk1+ embryos treated with DMSO, 50 µM BIO, and 100 µM BIO for 6 hours at 2 hphs. Red line indicates normalized expression levels in DMSO treated non-transgenic controls with ef1alpha as an internal control. BIO induces expression of Wnt targets in non-transgenic controls and rescues expression in dntcf3+ and dkk1+ embryos (G,H). (TIF) [file pgen.1004479.s003.tif]

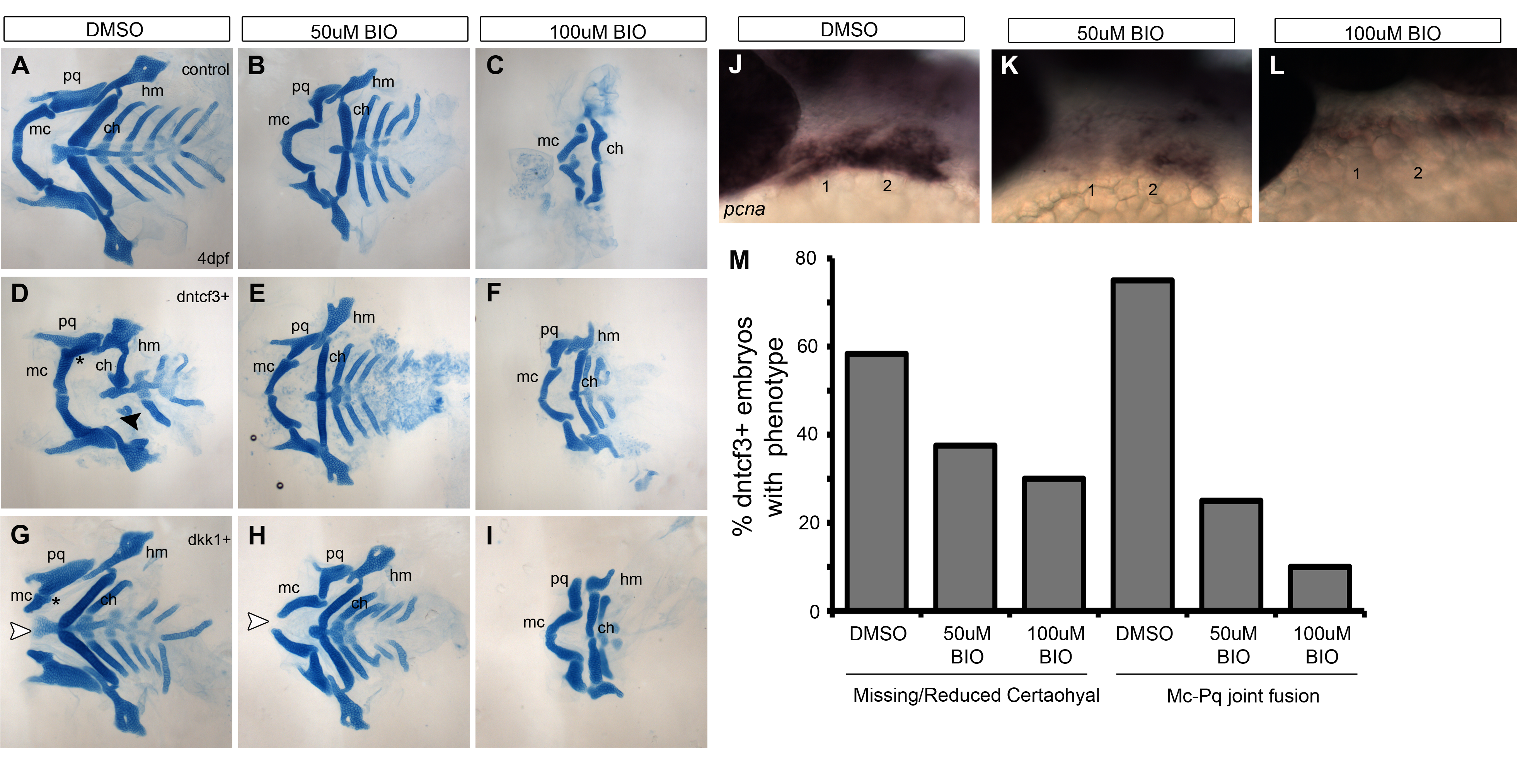

Supplement: Figure S4 — BIO rescues D-V defects in dntcf3+ and dkk1+ embryos. (A–I) Dissected, flat mounted alcian-stained cartilage at 4 dpf, ventral views, anterior to the left; non-transgenic control (top row), dntcf3+ (middle row) and dkk1+ (bottom row), untreated (A, D, G), or treated with 50 µM (B, E, H) or 100 µM (C, F, I) BIO for 6 hours. White arrowheads in G-H indicate Mc clefting, and black arrowheads indicate missing Ch in D. Asterisks in D and G indicate Mc-Pq joint fusions. High concentrations of BIO reduce overall cartilage size and cause specific loss of dorsal cartilages (C). BIO treatments rescue Mc-Pq joint fusions in both dntcf3+ and dkk1+ embryos (E, F, H, I, M) as well as Ch in dntcf3+ embryos (E, F, M). High BIO concentrations rescue Mc clefting in dkk1+ embryos (I). (J–L) Whole mount ISH for pcna in control embryos treated with DMSO, 50 µM BIO, and 100 µM BIO. (M) Histogram quantifying the percentage of dntcf3+ embryos showing Ch loss or Mc-Pq joint fusion in response to BIO treatments. Abbreviations: Ch, ceratohyal; Hm, hyomandibular; Mc, Meckel's; Pq, palatoquadrate. (TIF) [file pgen.1004479.s004.tif]

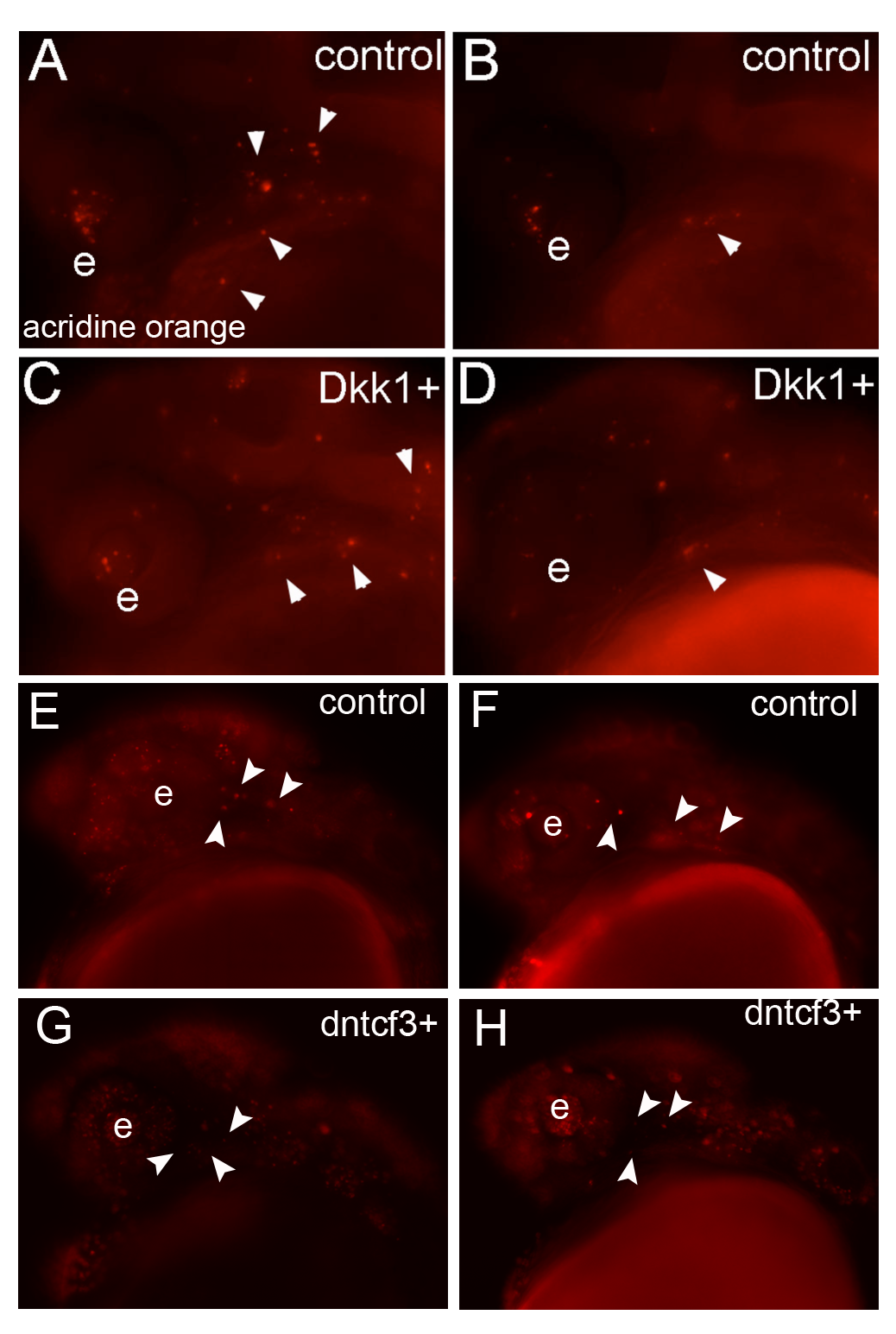

Supplement: Figure S5 — Cell death is not increased in dkk1+ and dntcf3+ embryos. (A–H) Lateral views of live acridine orange staining at 6 hphs in control (A, B, E, F), dkk1+ (C, D), and dntcf3+ (G, H) embryos. White arrowheads indicate apoptotic cells. Abbreviations: e, eye. (TIF) [file pgen.1004479.s005.tif]

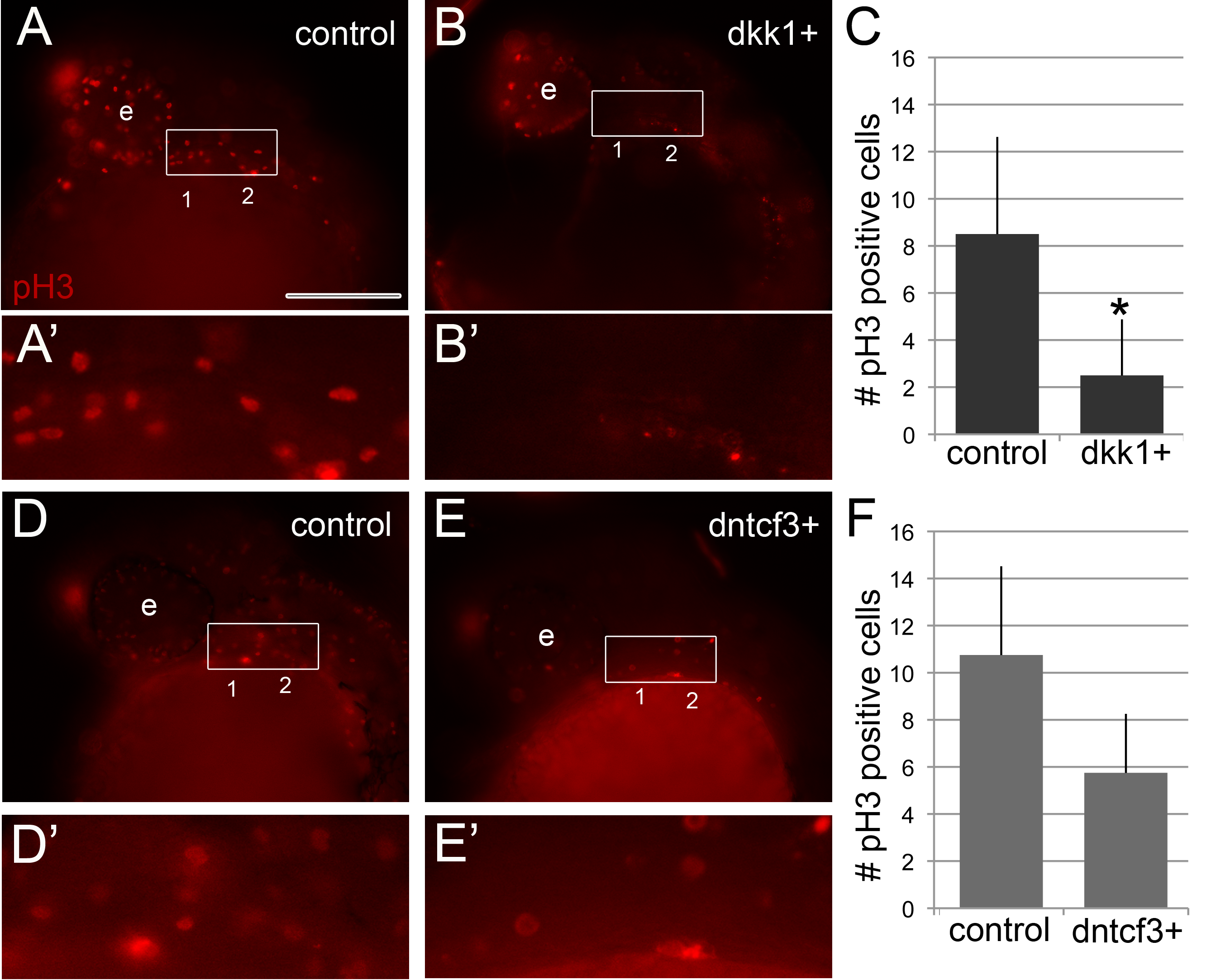

Supplement: Figure S6 — Arch proliferation defects in dkk1+ and dntcf3+ embryos. (A, B, D, E) Anti-phosphoHistone3 (pH 3) staining in controls (A, A′) and dkk1+ (B, B′) embryos stained 3 hphs and in controls (D, D′) and dntcf3+ (E, E′) embryos stained 4 hphs; lateral views, anterior to the left. (A′, B′, D′, E′). Enlargements of boxed areas encompassing presumptive first and second arches used for quantification. (C, F) Histograms quantifying numbers of pH 3 positive cells in the pharyngeal region of control and dkk1+ embryos, p<0.05 (C) and dntcf3+ embryos, p = 0.06 (F). Scale bar: 100 µm. (TIF) [file pgen.1004479.s006.tif]

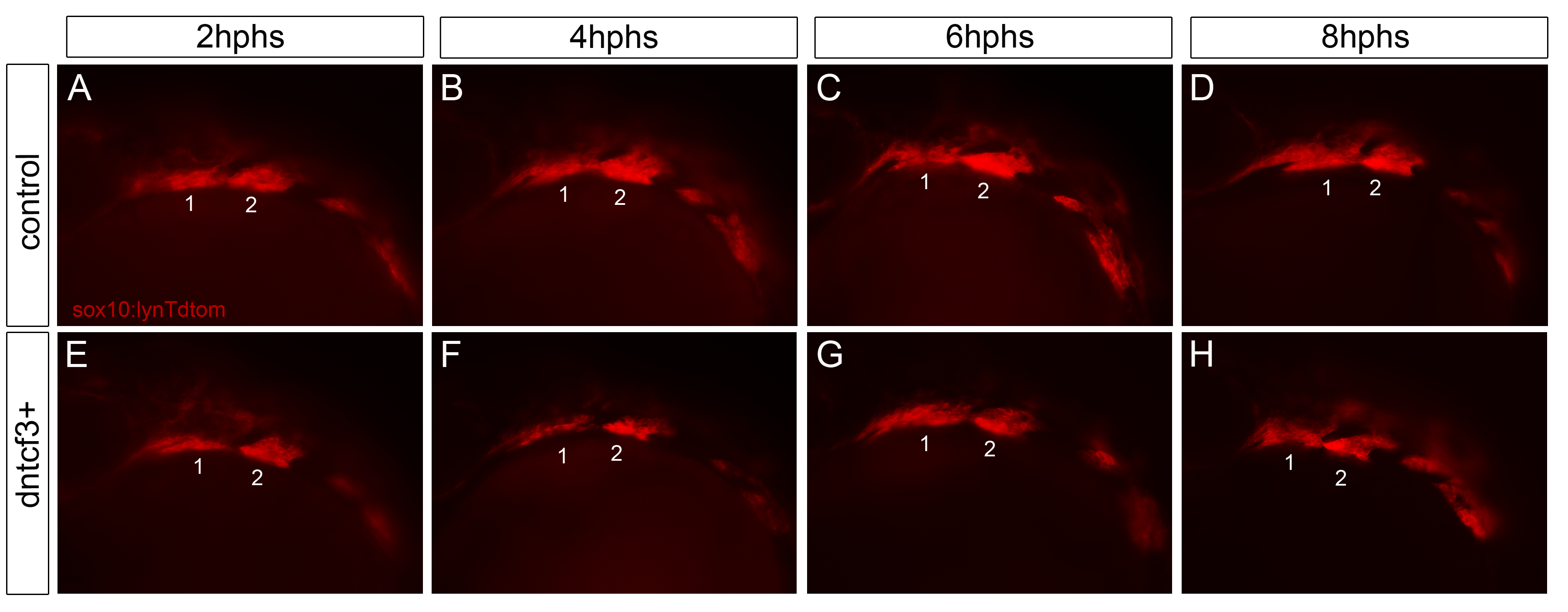

Supplement: Figure S7 — Neural crest migration in dntcf3+ embryos. (A–H) Live images of Tg(sox10:lyn-Tdtomato) fluorescence in cranial neural crest cells in the arches in control (A–D) and dntcf3+ (E–H) embryos at 2 hour intervals post heatshock, lateral views, anterior to the left. (TIF) [file pgen.1004479.s007.tif]

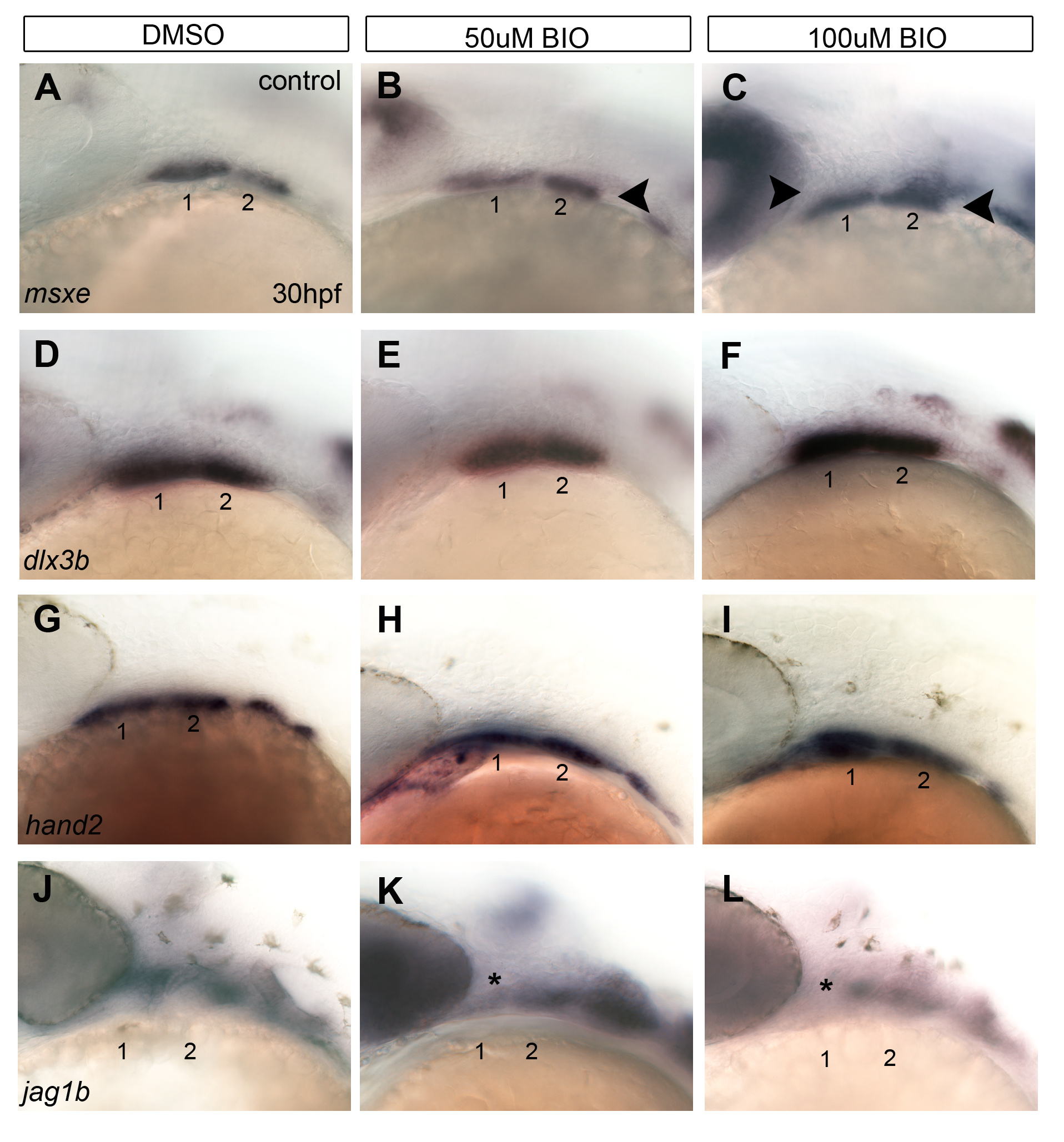

Supplement: Figure S8 — BIO disrupts dorsal-ventral patterning gene expression. (A–L) Lateral views of whole mount ISH for D-V patterning genes in embryos treated with DMSO (A, D, G, J), 50 µM BIO (B, E, H, K), and 100 µM BIO (C, F, I, L) for 6 hours at 24 hpf. With increasing concentrations of BIO, expression of the ventral-intermediate gene msxe expands dorsally (B, C) (black arrowheads). In response to BIO treatments, expression domains of hand2 (H–I) and dlx3b (E–F) only expand slightly. Expression of the dorsally restricted jag1b is reduced (asterisk) in the first and second arches of BIO treated embryos (K–L). (TIF) [file pgen.1004479.s008.tif]

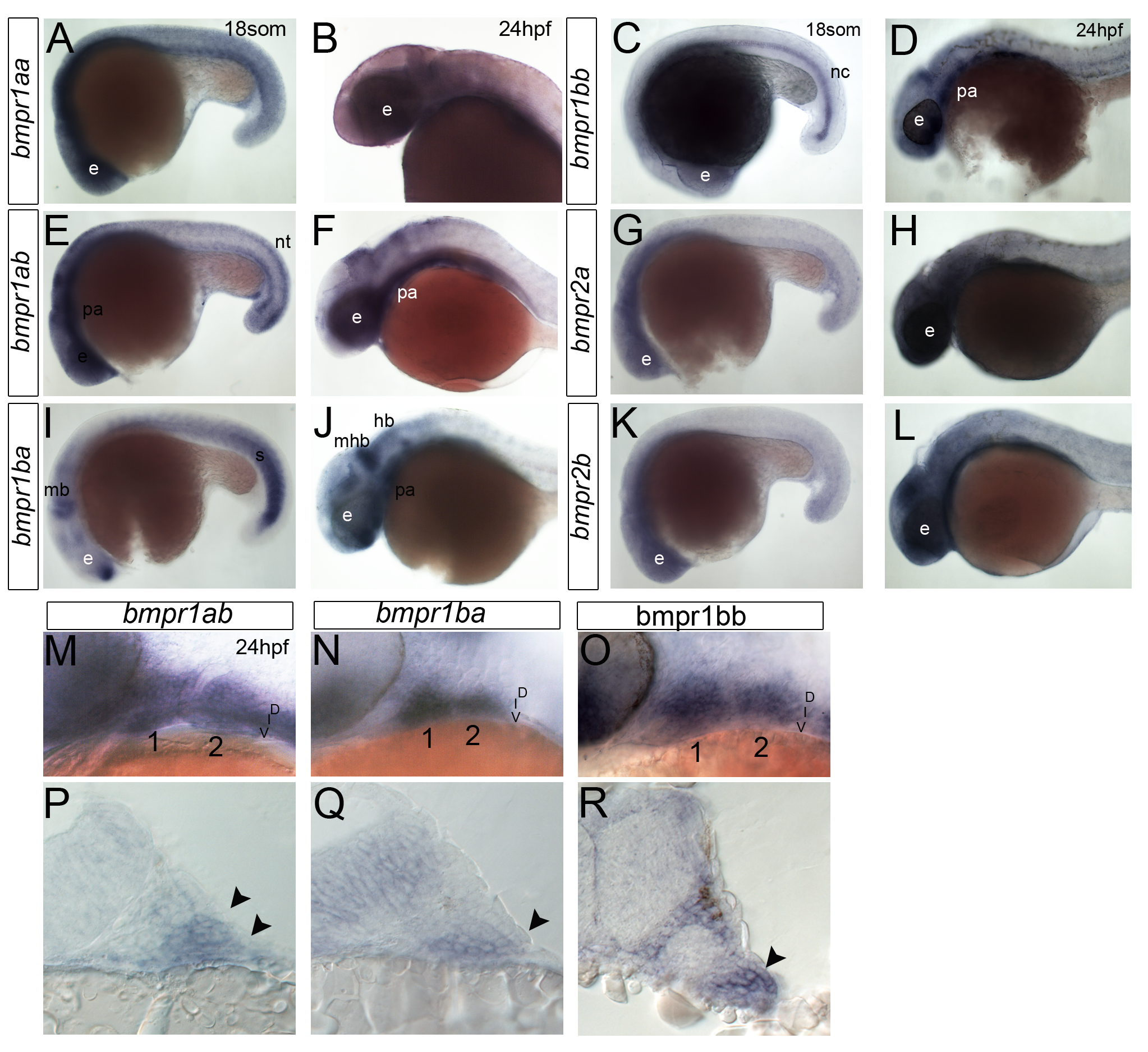

Supplement: Figure S9 — Bmp receptor expression during pharyngeal arch development. (A–O) Whole mount ISH for bmpr1aa (A, B), bmpr1bb (C, D, O), bmpr1ab (E, F, M), bmpr2a (G, H), bmpr1ba (I, J, N), and bmpr2b (K, L), lateral views, anterior to the left. (P–R) Transverse sections through 24 hpf embryos showing expression of bmpr1ab (P), bmpr1ba (Q), and bmpr1bb (R) in neural crest cells of the pharyngeal arches. Arrowheads indicate ventral restriction of bmpr1ab (P) and bmpr1bb (Q) expression compared with bmpr1ab (R). Abbreviations: e, eye; hb, hindbrain; mhb, mid-hindbrain boundary; nc, notocord; nt, neural tube; pa, pharyngeal arches; s, somites. (TIF) [file pgen.1004479.s009.tif]

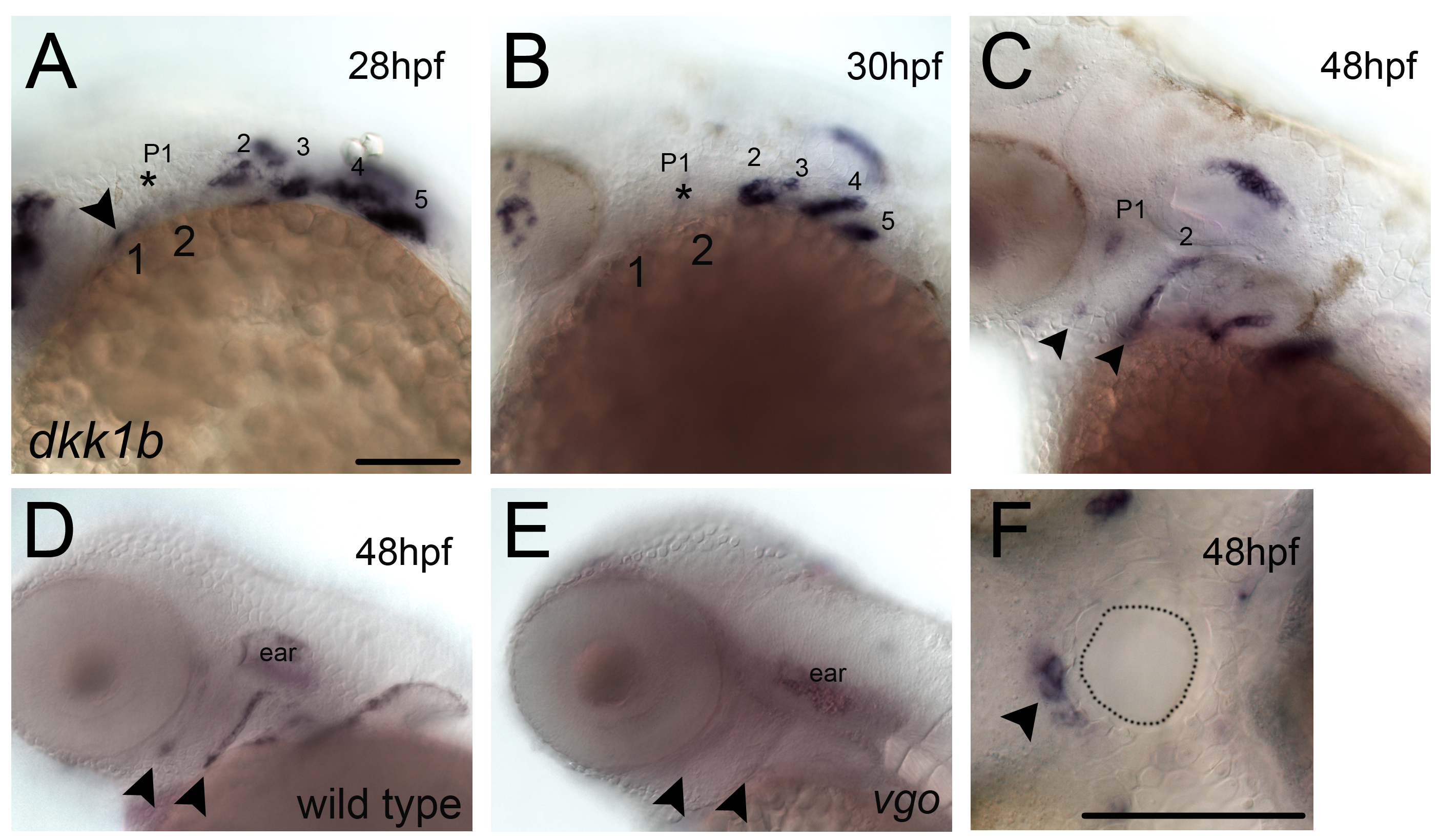

Supplement: Figure S10 — Dkk1 expression in pharyngeal endoderm. (A–F) Whole mount ISH for dkk1b in wild type (A–D, F) and van gogh (vgo) (E) embryos, (A–E) lateral and (F) ventral views, anterior to the left. Arrowhead in A indicates expression in the first arch. Asterisks in A and B indicate the first pharyngeal pouch. Arrowheads in C-E indicate the first and second pouches. (F) Ventral view at 48 hpf showing dkk1b expression in the oral ectoderm (arrowhead) surrounding the mouth opening (dotted line). Scale bar: 100 µm. (TIF) [file pgen.1004479.s010.tif]

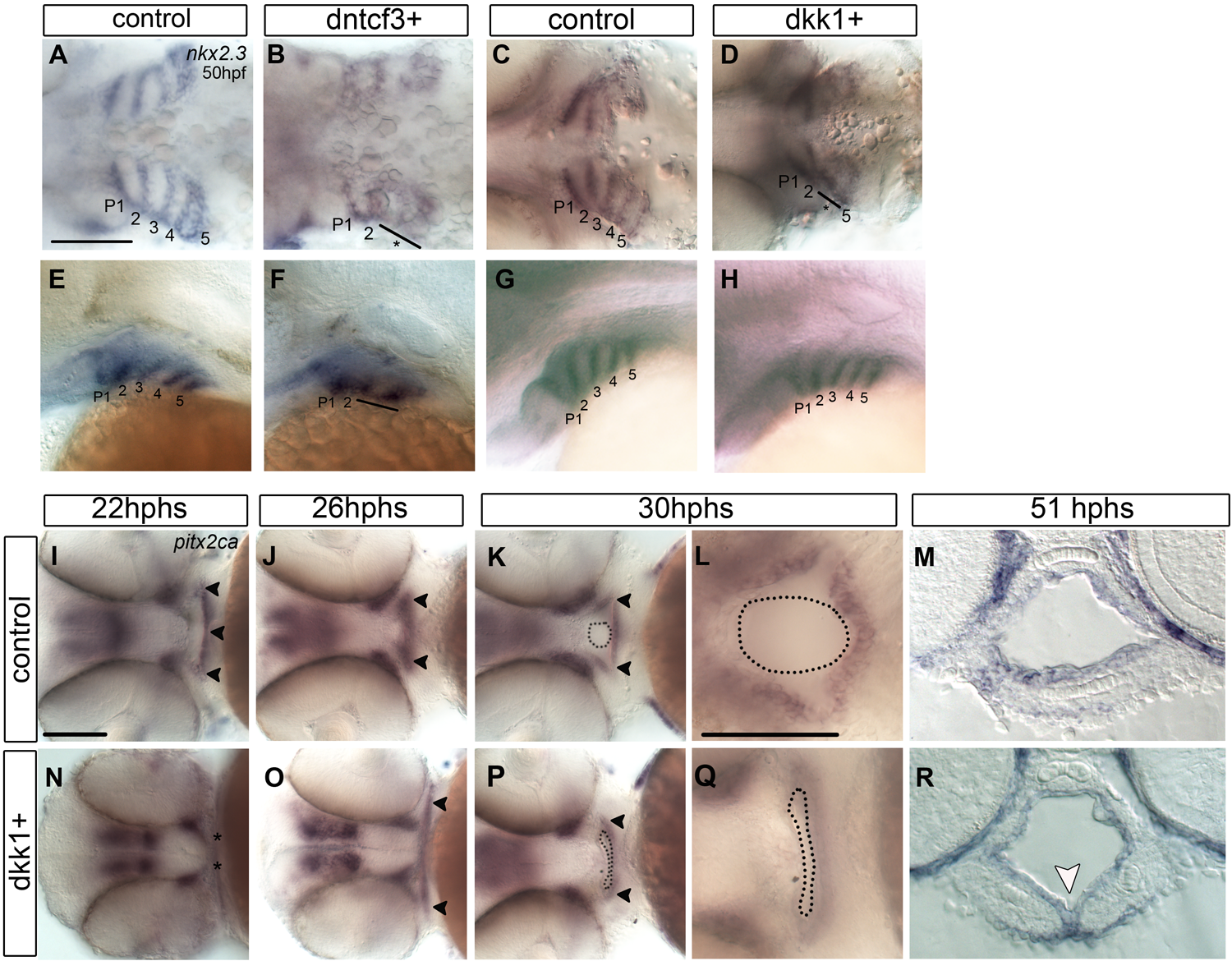

Supplement: Figure S11 — Defects in endoderm and oral ectoderm in dkk1+ embryos (A–L, N–Q) Whole mount ISH, anterior to the left. (A–H) Dorsal and lateral views showing nkx2.3 expression in pharyngeal pouch endoderm at 50 hpf in controls (A, C, E, G), dntcf3+ (B, F), and dkk1+ (D, H) embryos. Normal pouches are numbered. Asterisks and black lines indicate defective pouches. (I–L, N–Q) Ventral views showing pitx2ca expression in control (I–L) and dkk1+ (N–Q) embryos. (M, R) Transverse sections of the mouth showing pitx2ca expression in control (M) and dkk1+ (R) embryos at 51 hpf. Arrowheads indicate expression in oral ectoderm. Dotted lines in K, L, P and Q indicate the mouth opening. White arrowhead indicates a ventral midline fold in the mouth. Scale bars: 100 µm. (TIF) [file pgen.1004479.s011.tif]
